# Remifentanil reduces post-induction hypotension compared to fentanyl in thoracoscopic esophagectomy: a retrospective cohort study

**DOI:** 10.3389/fphar.2025.1660228

**Published:** 2025-10-09

**Authors:** Jin Zhang, Chen Zhao, Hao Sun, Youming Deng, Guangfu Qian, Shibing Teng, Baoxin Wang

**Affiliations:** ^1^ Department of Anesthesiology, The Second Hospital of Nanjing, Nanjing, Jiangsu, China; ^2^ Department of Anesthesiology, Suzhou Xiangcheng People’s Hospital, Suzhou, Jiangsu, China; ^3^ Department of Thoracic Surgery, Suzhou Xiangcheng People’s Hospital, Suzhou, Jiangsu, China; ^4^ Department of Otolaryngology, Shanghai General Hospital, Shanghai Jiao Tong University School of Medicine, Shanghai, China

**Keywords:** post-induction hypotension, anesthesia, induction, remifentanil, fentanyl, thoracoscopic esophagectomy

## Abstract

**Background:**

Post-induction hypotension (PIH) is a common complication associated with anesthesia, particularly in high-risk groups, such as elderly, malnourished patients with multiple comorbidities undergoing thoracoscopic esophagectomy. The selection of induction agents plays a significant role in influencing hemodynamic stability. However, there is a lack of comprehensive comparative data regarding the impact of different opioid agents on PIH.

**Methods:**

This retrospective cohort study included 289 patients undergoing thoracoscopic esophagectomy, who received etomidate combined with either fentanyl (Fentanyl group) or remifentanil (Remifentanil group) for anesthesia induction. A logistic regression model was used to examine the association between the induction regimen and PIH. Confounding factors were adjusted using a directed acyclic graph, and least absolute shrinkage and selection operator (LASSO) regression was employed to select covariates, ensuring robustness of the primary outcome analysis. Hemodynamic changes in systolic blood pressure, mean arterial pressure, and heart rate during the first 15 min post-induction were analyzed using generalized estimating equations to account for correlated observations. Subgroup analyses were performed for key clinical subgroups.

**Results:**

Among 289 patients analyzed, the incidence of PIH was significantly lower in the Remifentanil group compared to the Fentanyl group (23.7% vs. 42.3%, *P* = 0.001; adjusted odds ratio (OR) = 0.42, 95% confidence interval (CI): 0.25–0.73). Sensitivity analysis using LASSO-selected covariates yielded consistent results (adjusted OR = 0.41, 95% CI: 0.22–0.69, *P* = 0.001). Bradycardia occurred more frequently with remifentanil (11.9% vs. 4.5%, *P* = 0.03), whereas post-intubation hypertension and phenylephrine use were higher in the fentanyl group. No significant differences were observed in cardiovascular complications or postoperative hospital stay. Subgroup analyses revealed no significant effect modification across age, hemoglobin, or albumin levels. Remifentanil was also associated with more stable hemodynamics, including attenuated systolic blood pressure decline and lower variability during the first 15 min post-induction.

**Conclusion:**

Remifentanil-based general anesthesia induction reduces the risk of PIH and enhances hemodynamic stability in patients undergoing thoracoscopic esophagectomy.

## Introduction

Esophageal cancer is one of the most prevalent malignancies globally, with a particularly high incidence among the elderly population (Baiu and Backhus, 2020). Patients with this condition often suffer from malnutrition, chronic comorbidities, and the added burden of neoadjuvant chemotherapy (Chou et al., 2022; Ewer and Ewer, 2015; Harada et al., 2025; Thomson et al., 2018). These features reduce tolerance to anesthesia during induction and increase anesthesia risk. Post-induction hypotension (PIH) is a common complication of anesthesia induction. Across studies, the incidence of PIH ranges from 18% to 56%, with severe hypotension in about 35% of older adults (Terwindt et al., 2024; Schonberger et al., 2022). Established risk factors for PIH include advanced age, male sex, low body mass index (BMI), cardiovascular comorbidities, and preoperative antihypertensive therapy (Reich et al., 2005; Südfeld et al., 2017; Chen et al., 2021; Hoppe et al., 2022). Patients undergoing esophagectomy often present with several of these characteristics. Furthermore, evidence in esophageal cancer suggests that neoadjuvant chemotherapy impairs cardiopulmonary reserve (Navidi et al., 2018; Weaver and Patey, 2025), potentially increasing the risk of PIH. Therefore, this patient population should be considered a composite high-risk group. PIH is closely linked to adverse outcomes such as postoperative cardiovascular and cerebrovascular complications, renal dysfunction, and delayed recovery (Green and Butler, 2016; Mathis et al., 2020; Sessler et al., 2019; Walsh et al., 2013). Preventing PIH is therefore a critical goal in optimizing anesthesia induction strategies.

Etomidate provides rapid onset, short duration, and minimal cardiovascular depression, and is frequently chosen for induction in patients at risk of hemodynamic instability, particularly those with significant cardiovascular disease or limited cardiac reserve (Valk and Struys, 2021). It is commonly combined with fentanyl, which has rapid onset but an intermediate duration after a single bolus (Shafer and Varvel, 1991). However, a prospective observational study of 90 adult elective surgical patients reported that induction with etomidate combined with fentanyl resulted in a PIH incidence of approximately 47%, assessed within 10 min after intubation. Importantly, this cohort represented a relatively high-risk population, with more than half of the patients having preexisting cardiovascular disease (Zhang and Critchley, 2016). This high rate may be partly explained by fentanyl’s relatively long duration of action, which may extend into the interval between intubation and skin incision, an early low-stimulation phase vulnerable to hypotension (Südfeld et al., 2017; Reich et al., 2005). In contrast, remifentanil, an ultra-short-acting μ-opioid receptor agonist, has a rapid onset, predictable metabolism, and is unaffected by hepatic or renal function (Glass et al., 1993; Egan, 1995), making it theoretically better suited for precise hemodynamic control during anesthesia induction. Comparative studies under propofol-based induction have yielded heterogeneous hemodynamic results (Joshi et al., 2002; Twersky et al., 2001; Manukyan et al., 2025). By contrast, under etomidate induction, direct head-to-head comparisons of remifentanil versus fentanyl are scarce. Most available reports examined intubation responses or etomidate-induced myoclonus, lacked PIH as a prespecified endpoint, or assessed only very short observation windows (Ko et al., 2013; Kelsaka et al., 2006; Jiang et al., 2023; Hao et al., 2025).

To address this gap, this retrospective cohort study aims to compare the incidence of PIH and peri-induction hemodynamic changes between these two induction regimens, providing clinical evidence to inform anesthetic decisions for such high-risk surgical patients.

## Methods

### Study population

This retrospective cohort study was conducted by the Department of Anesthesiology at The Second Hospital of Nanjing, China, and was approved by the Institutional Ethics Committee (Approval No. 2025LSky033). To assess the hemodynamic effects of different anesthesia induction regimens, we retrospectively analyzed the clinical records of patients who underwent thoracoscopic esophagectomy for esophageal cancer between February 2022 and February 2025. Surgical candidacy was determined by the thoracic surgical team using guideline-based criteria. Indications were resectable disease according to the American Joint Committee on Cancer 8th-edition clinical tumor–node–metastasis (TNM) staging (typically T1–T3 or selected T4a, N0–N2, M0) with adequate physiological reserve. Contraindications included distant metastasis (M1), unresectable T4b, or prohibitive medical risk such as American Society of Anesthesiologists (ASA) classification IV–V. Patients meeting these contraindications were not scheduled for thoracoscopic esophagectomy at our institution. Inclusion criteria were adult patients undergoing elective thoracoscopic esophagectomy under general anesthesia with postoperative pathology confirming primary esophageal cancer. Exclusion criteria were (i) pre-induction thoracic paravertebral block or epidural anesthesia that could affect early peri-induction hemodynamics, (ii) incomplete records (missing exposure status, key preoperative variables, or insufficient continuous hemodynamic data within the 15-min peri-induction window), and (iii) an unexpected difficult airway during induction leading to failure to complete standard induction or conversion to awake intubation.

### Anesthesia management

According to the hospital information system records, all patients fasted for at least 8 hours and refrained from drinking water for 2 hours prior to surgery. For those scheduled for afternoon procedures, 500 mL of crystalloid fluid was typically administered preoperatively, in line with standard institutional practice. Anesthesia data were obtained from the anesthesia information system. Patients were classified into a fentanyl group or a remifentanil group according to the opioid used for induction. Drug doses and administration sequence followed institutional protocols.

Fentanyl group: Induction consisted of etomidate 0.2–0.3 mg/kg combined with fentanyl 2–4 μg/kg, followed by vecuronium 0.1 mg/kg for neuromuscular blockade; remifentanil was not given during induction.

Remifentanil group: Induction consisted of etomidate 0.2–0.3 mg/kg combined with remifentanil 1.0–2.0 μg/kg, followed by vecuronium 0.1 mg/kg for neuromuscular blockade.

Anesthesia maintenance: After tracheal intubation, anesthesia was maintained with sevoflurane and a continuous remifentanil infusion in both cohorts. The infusion was titrated to hemodynamic responses. After skin incision, the rate was usually increased to cover surgical stimulation, and supplemental fentanyl was administered as required.

### Data collection

We prespecified the baseline variables on clinical grounds and obtained data from the electronic medical record, the anesthesia information system, and the electronic prescribing system. Baseline variables included age; sex; BMI; ASA classification; serum albumin (Alb) and hemoglobin (Hb); comorbidities such as hypertension, diabetes, coronary artery disease, and chronic obstructive pulmonary disease (COPD); use of oral antihypertensive medications including angiotensin II receptor blocker (ARB), angiotensin-converting enzyme inhibitor (ACEI), calcium channel blocker (CCB), and beta blocker; and receipt of neoadjuvant chemotherapy. Malnutrition was defined as present if any of the following were met: BMI <18.5 kg/m^2^, Alb ≤35 g/L, or Hb ≤ 100 g/L. Comorbidity was defined as having one or more of the following diagnoses: hypertension, diabetes, coronary artery disease, or COPD. Oncologic and surgical variables were obtained from surgical records and operative notes, including the surgical approach (thoracoscopic McKeown or Ivor Lewis esophagectomy) and the extent of lymphadenectomy (two-field or three-field) as documented by the surgical team. Hemodynamic measurements during the first 15 min after induction, namely, systolic blood pressure (SBP), mean arterial pressure (MAP), and heart rate (HR), were obtained from the anesthesia information system under radial arterial monitoring at 0, 5, 10, and 15 min. Preoperative laboratory values were taken from tests performed within 7 days before surgery. Medication use was taken from the medication list on the day of surgery. Baseline vital signs refer to preinduction readings recorded in the operating room.

### Definition of post-induction hypotension

Given the lack of consensus on the definitions of post-induction and intraoperative hypotension, we referred to several large-scale studies (Bijker et al., 2007; Futier et al., 2017; Salmasi et al., 2017). PIH was defined as the occurrence of SBP <90 mmHg or a ≥30% decrease from baseline within 15 min after induction; fulfillment of either criterion on a single occasion was sufficient for classification. For sensitivity analyses, alternative definitions based on MAP were applied, namely, MAP <65 mmHg or a ≥20% decrease from baseline.

### Primary and secondary outcomes

The primary outcome of this study was post-induction hypotension. Secondary outcomes included bradycardia, defined as HR < 50 beats per minute (bpm), and post-intubation hypertension, defined as a greater than 20% increase in SBP (Yokose et al., 2016), the use of vasopressors during the perioperative period, the length of postoperative hospital stay, and the occurrence of severe cardiovascular complications, defined as major events like postoperative cerebral infarction and myocardial infarction.

### Statistical analysis

Normality was assessed using the Shapiro-Wilk test and visual inspection of histograms. Continuous variables are presented as mean ± standard deviation (SD) for normally distributed data, or as median [interquartile range (IQR)] for non-normally distributed data. For group comparisons of continuous variables, independent t-tests were used for normally distributed data, while Mann-Whitney U tests were applied for non-normally distributed data. Categorical variables are expressed as frequencies and percentages. Group comparisons of categorical variables were made using chi-square tests or Fisher’s exact tests. Odds ratios (ORs) and 95% confidence intervals (CIs) for proportions were estimated using logistic regression to assess the association between anesthetic induction regimens and PIH.

The primary outcome’s multivariable logistic regression model was developed based on a causal framework informed by clinical expertise, relevant literature, and a directed acyclic graph (DAG) (Figure 3) created using the Dagitty web application (http://www.dagitty.net/). The DAG was used to identify and adjust for key confounders, allowing for more robust causal inference (Textor et al., 2016). To assess robustness, sensitivity analyses included repeating the primary logistic model with a MAP-based PIH definition (MAP <65 mmHg or ≥20% decrease) using the same DAG-informed covariates, and applying least absolute shrinkage and selection operator (LASSO) logistic regression with 10-fold cross-validation for variable selection. The penalty parameter (λ) was chosen to minimize the cross-validation error (Supplementary Figure S1A). Variables with non-zero coefficients at λ min were considered informative; coefficient shrinkage and paths are shown in Supplementary Figure S1B.

The multivariable logistic regression equations were specified as follows, with PIH as the dependent variable and Logit P = $\ln\left(\frac{P}{1-P}\right)$

#### DAG-guided model

$$\text{logit }P\left(\text{PIH}=1\right)=\beta_{0}+\beta_{1}\cdot \text{Group }\left(\text{Remifentanil vs}.\text{ Fentanyl}\right)+\beta_{2}\cdot \text{Hypertension}+\beta_{3}\cdot \text{Age}+\beta_{4}\cdot \text{BMI}+\beta_{5}\cdot \text{Coronary artery disease}+\beta_{6}\cdot \text{Surgery }t\text{ime}$$

#### LASSO-selected model

$$\text{logit }P\left(\text{PIH}=1\right)=\beta_{0}+\beta_{1}\cdot \text{Group }\left(\text{Remifentanil vs}.\text{ Fentanyl}\right)+\beta_{2}\cdot \text{ARB}/\text{ACEI use}+\beta_{3}\cdot \text{CCB use}+\beta_{4}\cdot \text{Hypertension}+\beta_{5}\cdot N\text{eoadjuvant chemotherapy}+\beta_{6}\cdot \text{Sex}+\beta_{7}\cdot \text{Hb}$$

We applied prespecified diagnostic checks to both the DAG-guided and LASSO-selected multivariable logistic models, including assessments of logit linearity, multicollinearity, influential observations, separation, and overall model performance.

Subgroup analyses were conducted to explore potential effect modification based on clinically relevant characteristics and risk factors reported in the literature (Feng et al., 2021; Hu et al., 2021; Laurent et al., 2022). The predefined subgroups included age (>70 vs. ≤70 years), Hb level (≤100 vs. >100 g/L), and Alb level (≤35 vs. >35 g/L), which have been identified in previous studies as markers of frailty and increased perioperative risk. Due to small sample sizes in certain subgroups (such as patients with hypoalbuminemia, anemia, or those receiving neoadjuvant chemotherapy), Firth’s penalized likelihood logistic regression was applied for sensitivity analyses. This method was used to address rare events and minimize estimation bias, thereby preventing infinite estimates (Puhr et al., 2017).

Hemodynamic trends (SBP and HR) during the first 15 min following anesthesia induction were analyzed using generalized estimating equations (GEE) models. Measurements were taken at 0, 5, 10, and 15 min. The models included group, time, and their interaction (group × time) as fixed effects to assess temporal differences between anesthetic strategies. Confounders, identified using a DAG, were adjusted for in all models. SBP variability during the first 15 min after anesthesia induction was assessed using two indices: the coefficient of variation (CV) and average real variability (ARV) (Coccina et al., 2019; Hirsch et al., 2015), based on values recorded every 5 min. CV was calculated as the ratio of the standard deviation to the mean SBP and expressed as a percentage. ARV was defined as the mean of the absolute differences between consecutive SBP measurements. All statistical analyses were performed using R software (version 4.4.1; R Core Team, 2024) with the following packages: stats for normality tests, t-tests, chi-square tests, and logistic regression, glmnet for LASSO regression, geepack for GEE models, and ggplot2 for data visualization.

### Missing data handling

Arterial blood pressure measurements at 5-min intervals during the first 15 min post-induction were required for evaluating hemodynamic trends and variability. Patients with substantial missing Arterial blood pressure data (defined as having fewer than 3 valid measurements during this period) were excluded. Implausible values (e.g., SBP < 40 mmHg or > 300 mmHg), likely resulting from signal artifacts, were treated as missing and imputed using linear interpolation when only a single time point was affected. Cases with multiple or consecutive missing values were excluded from time-series and variability analyses. For baseline covariates used in multivariable models, missingness was minimal (<5%) and handled using a complete-case approach. No multiple imputation was performed.

## Results

**FIGURE 1:**
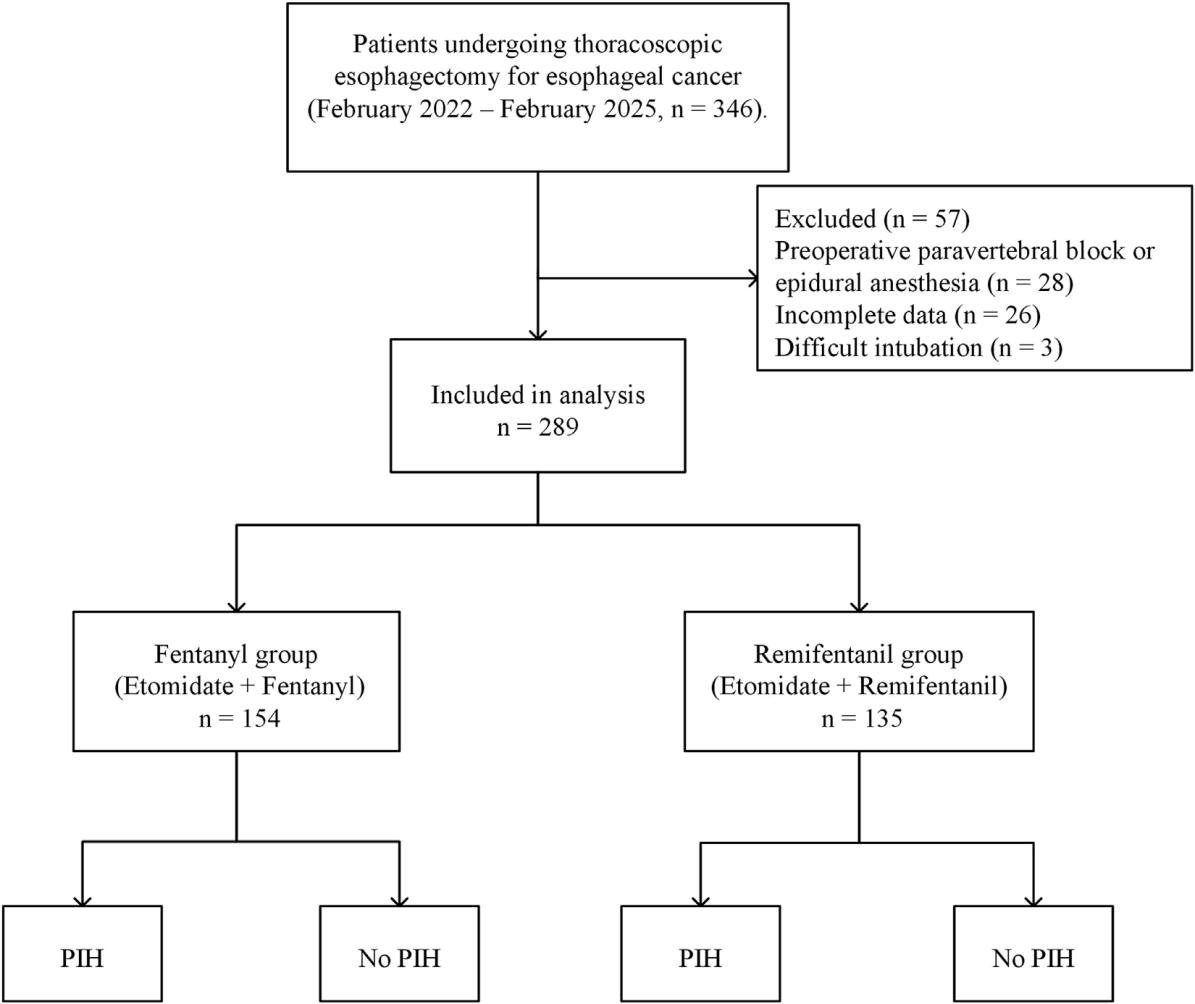
Flowchart of patient inclusion and exclusion. Flowchart showing the patient selection process, with 289 patients included after exclusions, divided into the fentanyl group and the remifentanil group, further categorized by the occurrence of PIH. Abbreviations: PIH, post-induction hypotension.

A total of 346 patients scheduled for thoracoscopic esophagectomy were initially screened through the hospital’s electronic medical record system. After applying the exclusion criteria, 57 patients were excluded: 28 had received preoperative paravertebral block or epidural anesthesia, 26 had incomplete perioperative or hemodynamic data, and 3 experienced difficult intubation precluding standard induction protocols. Consequently, 289 patients were included in the final analysis. Patients were categorized into two groups based on the induction opioid used: the Fentanyl group (n = 154) and the Remifentanil group (n = 135). A detailed flowchart of patient selection is presented in Figure 1. The two groups were overall comparable at baseline (Table 1).

**TABLE 1:** Baseline characteristics.

Characteristics	Overall	Fentanyl group	Remifentanil group	*P* Value
	(n = 289)	(n = 154)	(n = 135)	
Age (years), mean ± SD	68.7 ± 7.6	68.2 ± 7.1	69.4 ± 8.1	0.14
Sex, No. (%)				
Male	236 (81.7)	126 (81.8)	110 (81.5)	0.22
Female	53 (18.3)	28 (18.2)	25 (18.5)	
BMI (kg/m^2^), mean ± SD	23.0 ± 3.08	22.8 ± 2.9	23.2 ± 3.2	0.15
BMI < 18.5 kg/m^2^, No. (%)	21 (7.3)	10 (6.5)	11 (8.1)	0.65
Smoking, No. (%)	124 (42.9)	63 (40.9)	61 (45.2)	0.48
Alcohol use, No. (%)	89 (30.7)	47 (29.4)	42 (30.0)	0.98
ASA classification, No. (%)				
II	133 (46.0)	66 (42.9)	67 (49.6)	0.28
III	156 (54.0)	88 (57.1)	68 (50.4)	
Hypertension, No. (%)	137 (47.4)	74 (48.1)	63 (46.7)	0.91
COPD, No. (%)	28 (9.7)	17 (11.0)	11 (8.1)	0.43
Diabetes, No. (%)	23 (7.9)	14 (9.0)	9 (6.7)	0.52
Coronary artery disease, No. (%)	33 (11.4)	13 (8.4)	20 (14.8)	0.09
Any comorbidity	165 (57.1)	90 (58.4)	75 (55.6)	0.71
ARB/ACEI use, No. (%)	68 (23.5)	35 (22.7)	33 (24.4)	0.78
CCB use, No. (%)	75 (26.0)	36 (23.4)	39 (28.9)	0.35
Beta-blocker use, No. (%)	13 (4.5)	3 (1.9)	10 (7.4)	0.04
Diuretics use, No. (%)	16 (5.5)	12 (7.8)	4 (3.0)	0.12
Neoadjuvant chemotherapy, No. (%)	64 (22.1)	40 (26.0)	24 (17.8)	0.12
Surgical type, No. (%)				
McKeown	284 (98.3)	153 (99.4)	131 (97.0)	0.19
Ivor Lewis	5 (1.7)	1 (0.6)	4 (3.0)	
Lymph node dissection, No. (%)				
Two-field	276 (95.5)	150 (97.4)	126 (93.3)	0.15
Three-field	13 (4.5)	4 (2.6)	9 (6.7)	
Surgery time, No. (%)				
Morning	159 (55.0)	86 (55.8)	73 (54.1)	0.81
Afternoon	130 (45.0)	68 (44.2)	62 (45.9)	
Induction–incision time (min), median [IQR]	19.1 [17.6–20.7]	20.1 [18.6–21.8]	18.0 [16.5–19.4]	0.01
Alb (g/L), mean ± SD	40.6 ± 4.2	40.4 ± 4.1	40.7 ± 4.3	0.49
Alb ≤ 35 g/L, No. (%)	24 (8.3)	11 (7.1)	10 (7.4)	0.99
Hb (g/L), mean ± SD	126.1 ± 17.8	126.9 ± 16.3	125.2 ± 19.4	0.43
Hb ≤ 100 g/L, No. (%)	25 (8.7)	10 (6.5)	11 (11.1)	0.21
Malnutrition	58 (20.1)	29 (18.8)	29 (21.5)	0.68
SBP (mmHg), median [IQR]	149 [136.0–161.0]	150 [134.3–161.8]	148 [138.0–160.5]	0.07
MAP (mmHg), median [IQR]	97 [89.0–104.0]	99 [91.0–105.0]	94 [87.5–102.5]	0.03
HR (bpm), median [IQR]	73 [64.0–80.0]	73 [65.0–81.75]	72 [62.0–79.0]	0.12

Data are presented as mean ± SD, median [IQR], or No. (%). Malnutrition was defined as present if any of the following were met: BMI < 18.5 kg/m^2^, Alb ≤ 35 g/L, or Hb ≤ 100 g/L. Comorbidity was defined as having ≥1 of the following diagnoses: hypertension, diabetes, coronary artery disease, or COPD.

Abbreviations: SD, standard deviation; IQR, interquartile range; No., number; BMI, body mass index; ASA, american society of anesthesiologists; ARB, angiotensin II, receptor blocker; ACEI, angiotensin-converting enzyme inhibitor; CCB, calcium channel blocker; COPD, chronic obstructive pulmonary disease; Alb, albumin; Hb, hemoglobin; SBP, systolic blood pressure; MAP, mean arterial pressure; HR, heart rate; bpm, beats per minute.

**FIGURE 2:**
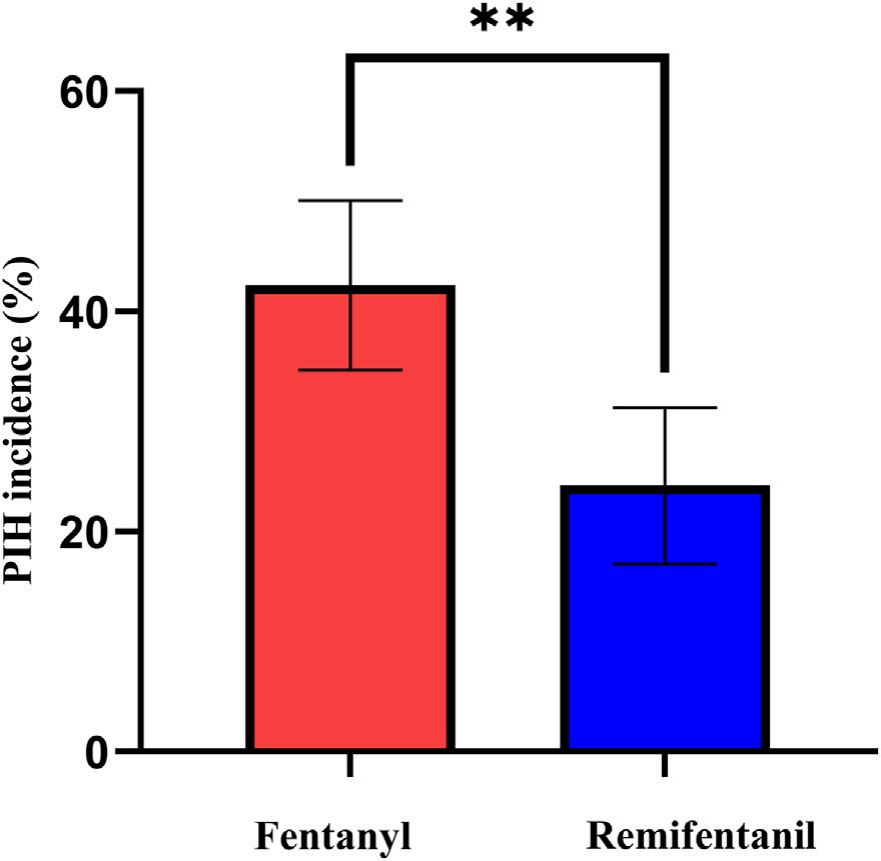
Incidence of PIH in the fentanyl and remifentanil groups. Incidence of PIH in the fentanyl and remifentanil groups. Bars represent the observed incidence (%), and error bars indicate 95% confidence intervals calculated by the Wilson score method. Asterisks: *P < 0.05, **P < 0.01, ***P < 0.001. Abbreviations: PIH, post-induction hypotension.

**FIGURE 3:**
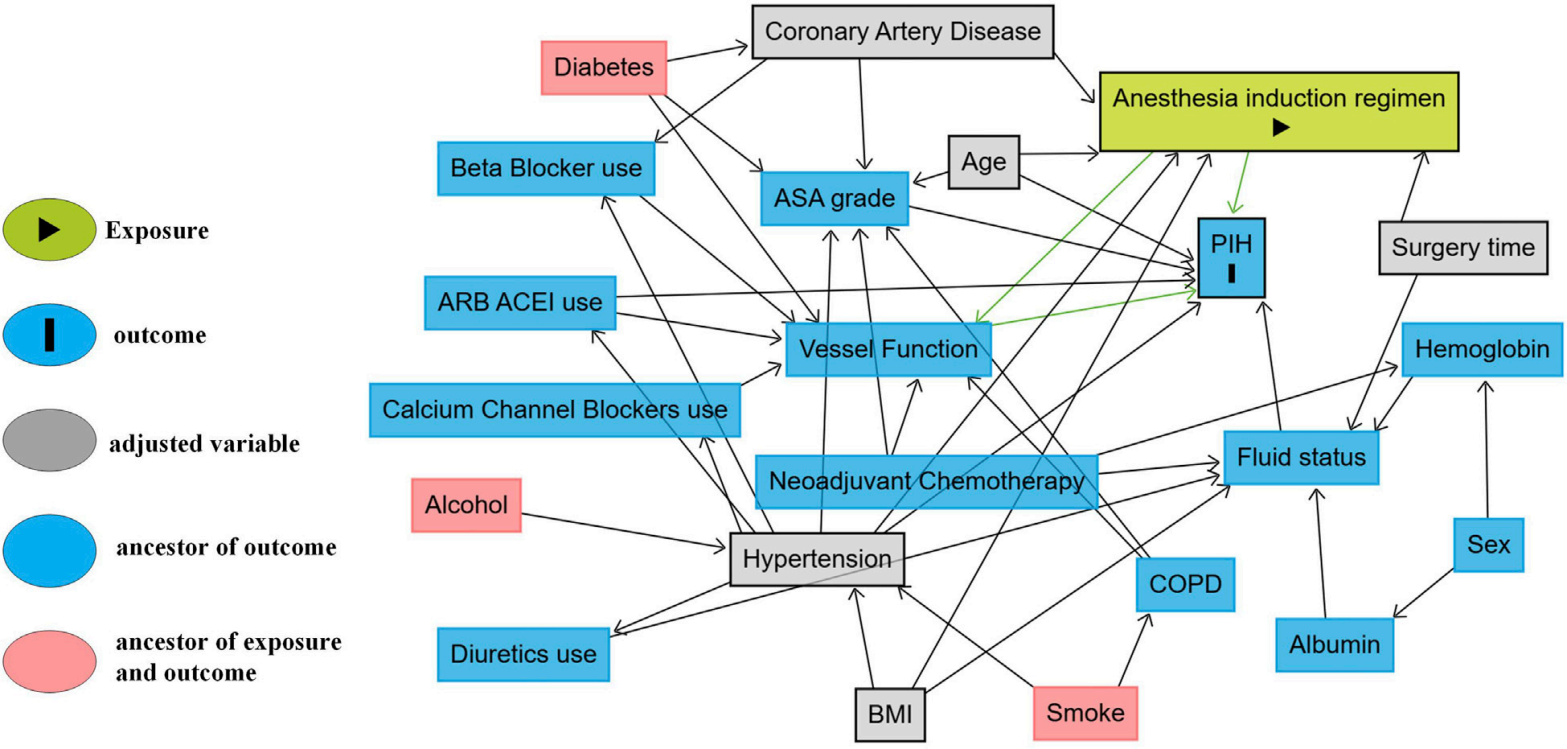
DAG illustrating potential causal pathways between anesthesia induction regimen and PIH. DAG of the assumed causal relation between the induction opioid (remifentanil vs. fentanyl) and PIH. Grey: minimal sufficient adjustment set (hypertension, surgery time [morning vs. afternoon], BMI, age, coronary artery disease). Blue: measured ancestors of the outcome not included in the adjustment set. Pink: putative common causes of exposure and outcome. Arrows denote assumed causal directions. Abbreviations: DAG, directed acyclic graph; PIH, post-induction hypotension; ASA, American Society of Anesthesiologists; BMI, body mass index; COPD, chronic obstructive pulmonary disease; ARB, angiotensin II receptor blocker; ACEI, angiotensin-converting enzyme inhibitor.

As shown in Figure 2 and Table 2, the incidence of PIH was lower with remifentanil than with fentanyl (23.7% vs. 42.3%; *P* = 0.001). The crude OR was 0.43 (95% CI 0.26–0.71; *P* = 0.001). After adjustment using the prespecified DAG-informed confounder set (Figure 3), the association remained (adjusted OR = 0.42 95% CI 0.25–0.73; *P* = 0.002; full outputs in Supplementary Table S1). A LASSO-selected model (Supplementary Figure S1A–B) yielded a similar estimate (adjusted OR = 0.41, 95% CI 0.22–0.69; *P* = 0.001; full outputs in Supplementary Table S2). All three estimates (crude, DAG-adjusted, LASSO-adjusted) are summarized in Table 2. Model diagnostics revealed no material violations such as logit nonlinearity, multicollinearity, or influential observations, and overall discrimination and calibration were acceptable. Details are provided in Supplementary Table S5 and Supplementary Figures S2, S3.

**TABLE 2:** Perioperative outcomes in the Fentanyl and Remifentanil groups.

Outcome	Fentanyl group (n = 154)	Remifentanil group (n = 135)	Difference or OR (95% CI)	*P* Value
Primary outcome				
PIH, No. (%)	65 (42.2)	32 (23.7)		
Crude OR			0.43 (0.26–0.71)	0.001
Adjusted OR (DAG model)			0.42 (0.25–0.73)	0.002
Adjusted OR (LASSO model)			0.41 (0.22–0.69)	0.001
Secondary outcome				
Hemodynamic safety				
Post-intubation hypertension, No. (%)	37 (24.0)	17 (12.6)	0.45 (0.24–0.85)	0.02
Bradycardia, No. (%)	7 (4.5)	16 (11.9)	2.86 (1.13–7.14)	0.03
Vasoactive agent use				
Ephedrine use, No. (%)	6 (3.9)	12 (8.9)	2.41 (0.88–6.60)	0.09
Phenylephrine use, No. (%)	33 (21.4)	11 (8.1)	0.33 (0.16–0.67)	0.002
Postoperative parameters				
Cardiovascular events, No. (%)	5 (3.2)	3 (2.2)	0.68 (0.16–2.89)	0.73
Length of postoperative hospital stay (days), median [IQR]	11.5 [10–14]	12.0 [11–14]	0 (-1–1)	0.24

Effect sizes are odds ratios (OR; remifentanil vs. fentanyl) with 95% confidence intervals (CI) for binary outcomes, and Hodges–Lehmann median differences (remifentanil − fentanyl) with 95% CI, for continuous outcomes.

DAG-adjusted model covariates: hypertension, surgery time (morning vs. afternoon), BMI, age, coronary artery disease. LASSO-adjusted model covariates: ARB/ACEI, use; CCB, use, hypertension, neoadjuvant chemotherapy, sex, hemoglobin (see Supplementary Figure S1; Supplementary Table S2). An OR < 1 indicates lower odds with remifentanil.

Abbreviations: IQR, interquartile range; No., number; OR, odds ratio; CI, confidence interval; PIH, post-induction hypotension; BMI, body mass index; ARB, angiotensin II, receptor blocker; ACEI, angiotensin-converting enzyme inhibitor; CCB, calcium channel blocker; DAG, directed acyclic graph; LASSO, least absolute shrinkage and selection operator.

In a sensitivity analysis using the MAP-based PIH definition, incidence remained lower with remifentanil (37.8% vs. 59.1%; *P* < 0.001; Supplementary Table S3). The corresponding crude and DAG-adjusted ORs were 0.45 (95% CI 0.29–0.69; *P* < 0.001) and 0.42 (95% CI 0.26–0.68; *P* = 0.004), respectively (Supplementary Table S4).

Bradycardia was more frequent with remifentanil (11.9% vs. 4.5%; remifentanil vs. fentanyl OR 2.86, 95% CI 1.13–7.14, P = 0.03). Post-intubation hypertension was less frequent with remifentanil (12.6% vs. 24.0%; remifentanil vs. fentanyl OR 0.45, 95% CI 0.24–0.85, P = 0.02). Phenylephrine was administered more often in the fentanyl group (21.4% vs. 8.1%; remifentanil vs. fentanyl OR 0.33, 95% CI 0.16–0.67, P = 0.002). Other peri-induction outcomes, including ephedrine use, cardiovascular complications, and length of postoperative stay, did not differ significantly between groups; see Table 2 for details.

**FIGURE 4:**
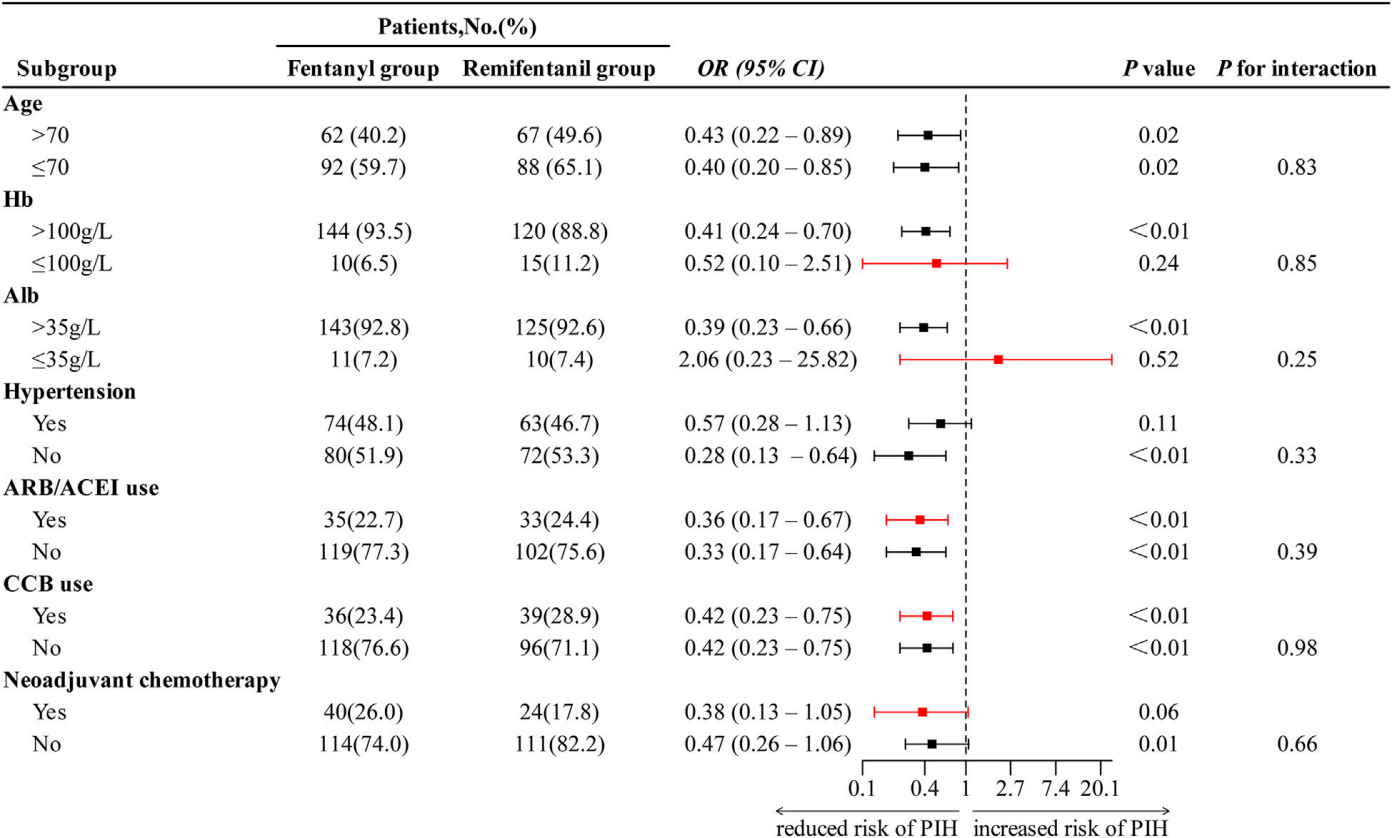
Subgroup analysis of the effect of anesthesia regimen on PIH. ORs with 95% confidence intervals are shown. Red markers indicate ORs estimated using Firth logistic regression due to small sample size or separation. OR < 1 indicates reduced risk of PIH in remifentanil group compared to fentanyl group. Abbreviations: PIH, post-induction hypotension; OR, odds ratio; CI, confidence interval; Hb, hemoglobin; Alb, albumin; ARB, angiotensin II receptor blocker; ACEI, angiotensin-converting enzyme inhibitor; CCB, calcium channel blocker.

Subgroup analyses showed no evidence of effect modification: across predefined subgroups of age, Hb, and Alb, the association between remifentanil compared with fentanyl and lower PIH risk did not differ (all P for interaction > 0.05; Figure 4). Although point estimates varied, including a reversed but imprecise and non-significant association in patients with hypoalbuminemia (albumin ≤ 35 g/L; OR 2.06, 95% CI 0.23–25.86; P = 0.52), the overall pattern remained consistent across subgroups.

**FIGURE 5:**
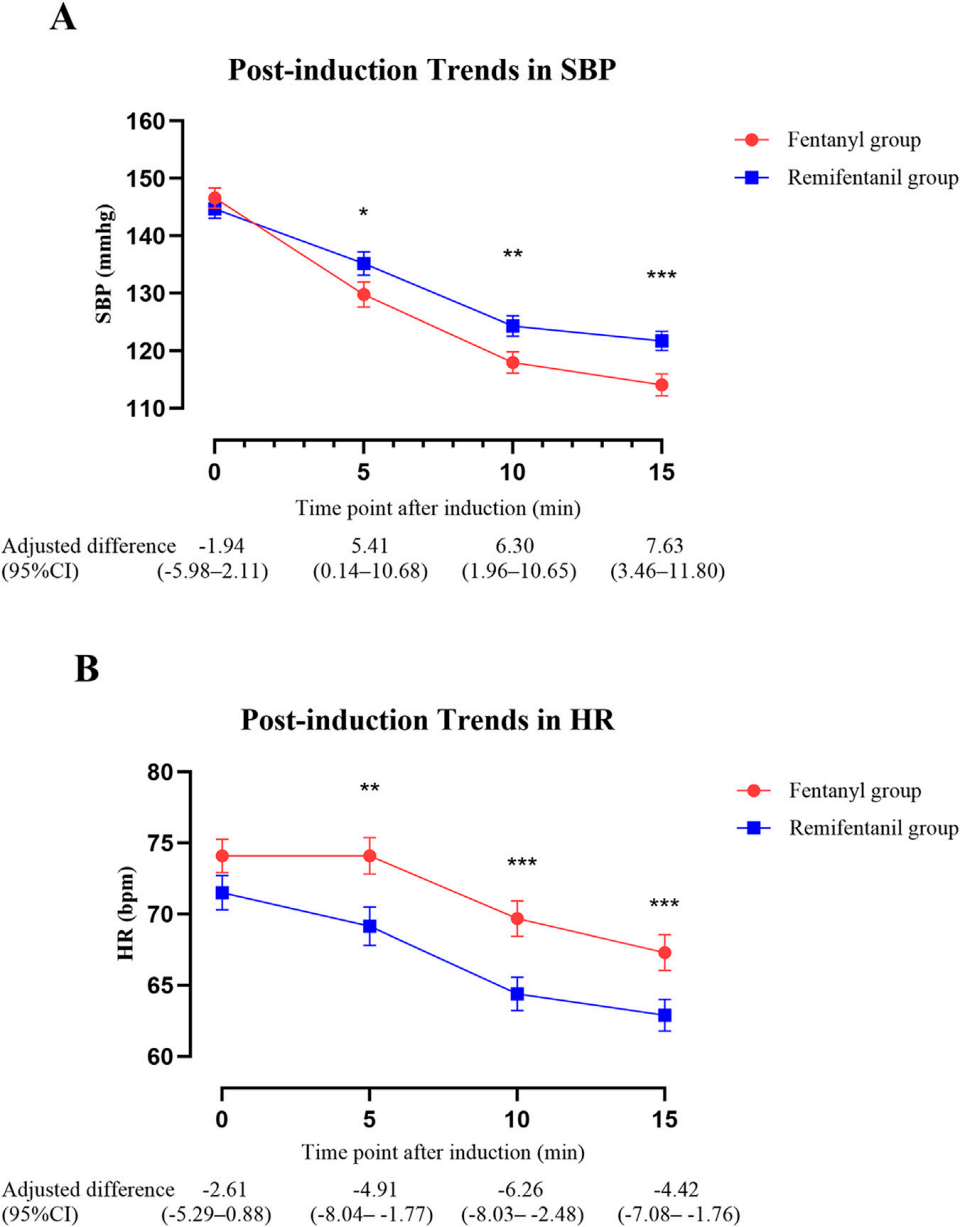
Trends in SBP and HR within 15 min after anesthesia induction. **(A)** SBP and **(B)** HR were analyzed using GEE adjusting for age, BMI, hypertension, surgery time (morning vs. afternoon), and coronary artery disease. Lines depict model estimated marginal means ± standard error by group at each time point. The table shows adjusted between-group differences (Remifentanil − Fentanyl) with 95% CIs at each time point. Asterisks mark time points with Bonferroni-adjusted significance: *P < 0.05, **P < 0.01, ***P < 0.001. Abbreviations: SBP, systolic blood pressure; HR, heart rate; BMI, body mass index; CI, confidence interval; GEE, generalized estimating equations; bpm, beats per minute.

Post-induction hemodynamic trends are presented in Figure 5. The GEE model was used to evaluate the overall group differences in HR and SBP trajectories during the first 15 min after induction. The analysis showed no significant group × time interaction for HR (*P* = 0.26), indicating similar chronotropic responses over time. However, pairwise comparisons with Bonferroni-adjusted *P* values revealed that HR was significantly lower in the remifentanil group at 5 min (adjusted difference, remifentanil minus fentanyl: −4.91 bpm, 95% CI -8.04 to −1.77; *P* = 0.002), 10 min (−6.26 bpm, 95% CI -8.03 to −2.48; *P* < 0.001), and 15 min (−4.42 bpm, 95% CI -7.08 to −1.76; *P* < 0.001) (Figure 5B). In contrast, SBP trends differed significantly between groups, with a significant group × time interaction (*P* = 0.003), and the remifentanil group demonstrated a more stable profile and an attenuated decline in SBP across the 15-min post-induction period. Specifically, SBP was significantly higher in the remifentanil group at 5 min (5.41 mmHg, 95% CI 0.14–10.68; *P* = 0.046), 10 min (6.30 mmHg, 95% CI 1.96–10.65; *P* = 0.005), and 15 min (7.63 mmHg, 95% CI 3.46–11.80; *P* < 0.001) (Figure 5A).

**FIGURE 6:**
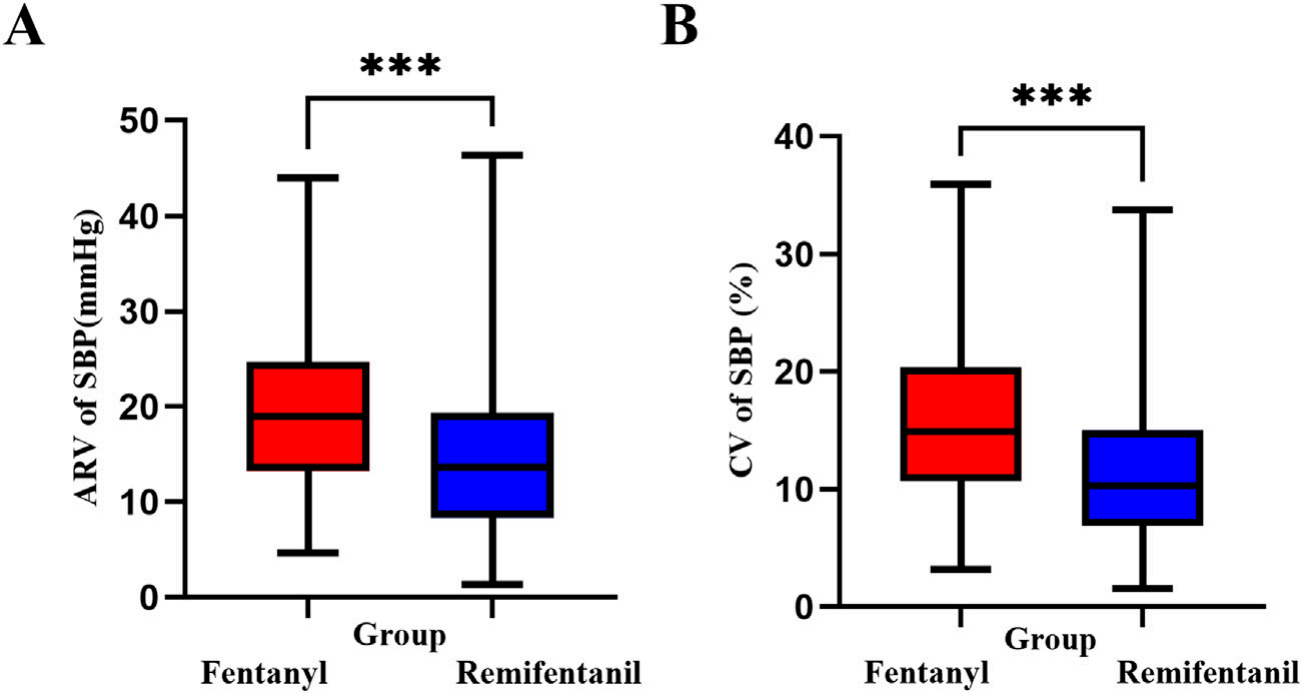
SBP variability within the first 15 min after anesthesia induction. **(A)** ARV (mmHg): Hodges–Lehmann median difference (remifentanil − fentanyl) = −4.67; two-sided Mann–Whitney *P* < 0.001. **(B)** CV (%): Hodges–Lehmann median difference = −4.26; *P* < 0.001. Boxes show median and IQR; whiskers extend to 1.5×IQR. Asterisks: *P < 0.05, **P < 0.01, ***P < 0.001. Abbreviations: SBP, systolic blood pressure; ARV, average real variability; CV, coefficient of variation; IQR, interquartile range.

As shown in Figure 6, SBP variability during the first 15 min following induction was significantly lower in the Remifentanil group, suggesting improved short-term hemodynamic stability. The CV of SBP (Figure 6B) was 10.3% (IQR: 8.1) in the Remifentanil group, compared to 14.9% (IQR: 9.4) in the Fentanyl group (*P* < 0.001). Similarly, the ARV (Figure 6A) was significantly lower with remifentanil (13.7 mmHg [IQR: 10.7] vs. 19.0 mmHg [IQR: 11.2]; *P* < 0.001), highlighting enhanced hemodynamic stability during the first 15 min following induction.

## Discussion

In this retrospective study of patients undergoing thoracoscopic esophagectomy, anesthesia induction with remifentanil combined with etomidate was associated with a significantly lower incidence of PIH compared to the conventional fentanyl–etomidate regimen. The remifentanil group also demonstrated greater hemodynamic stability. These findings suggest that remifentanil may be a more appropriate induction opioid for high-risk surgical patients. Despite the inherent limitations of retrospective designs, the use of DAG-informed confounder adjustment and LASSO sensitivity analysis enhances the robustness of our findings.

Perioperative hypotension lacks a single standard definition. A systematic review identified more than 100 definitions, and the reported incidence of PIH varies with the threshold, the observation window, the patient population, and the anesthetic regimen (Bijker et al., 2007; Salmasi et al., 2017). In our data, incidence differed when SBP rather than MAP criteria were applied. Regardless of the definition, remifentanil had lower PIH rates than fentanyl. These findings indicate that incidence should be interpreted in the context of the chosen definition, while the group comparison remains robust.

Although etomidate is often regarded as hemodynamically stable (Valk and Struys, 2021), the incidence of PIH remained high when it was combined with fentanyl. This likely reflects the physiological vulnerability of esophageal cancer patients, including malnutrition, chemotherapy exposure, altered autonomic tone, advanced age, hypertension, and frequent use of ARB/ACEI that blunt compensatory responses (Chou et al., 2022; Ewer and Ewer, 2015; Harada et al., 2025; Watson et al., 2021; Weaver and Patey, 2025; Hu et al., 2021). Mechanistically, both fentanyl and remifentanil are potent μ-opioid receptor agonists that suppress sympathetic outflow via Gi/o-coupled signaling (Williams et al., 2001; Pasternak and Pan, 2013), but their chemical structures and elimination pathways differ in clinically meaningful ways. Fentanyl, a 4-anilidopiperidine without an ester linkage, undergoes hepatic cytochrome P450 3A4 metabolism and exhibits a prolonged, context-sensitive half-time (Kuip et al., 2017). In susceptible patients, a single bolus may persist into the low-stimulation interval between induction and incision, potentially increasing the risk of hypotension. Remifentanil contains a hydrolyzable methyl ester and is rapidly degraded by nonspecific plasma/tissue esterases, yielding an ultrashort, context-insensitive half-time (Egan, 1995; Glass et al., 1996; Okano et al., 2025). This profile enables minute-to-minute titration during induction and intubation and allows rapid offset (Twersky et al., 2001), which can help hypotension resolve promptly once the infusion is reduced or stopped. Etomidate was routinely used across groups and provides hypnosis with minimal intrinsic analgesia; accordingly, induction analgesia was opioid-driven, and the observed post-induction blood pressure differences are more plausibly related to the opioid regimen (fentanyl vs. remifentanil) than to the hypnotic agent.

In published evidence, a prospective observational study of predominantly older, cardiovascular high-risk patients reported an early hypotension rate of nearly 47%. This study applied stricter thresholds (e.g., MAP <60 mmHg or >30% decrease) within a 10-min window after etomidate–fentanyl (Zhang and Critchley, 2016). Under etomidate induction and using the same PIH criteria as ours (SBP < 90 mmHg or ≥ 30% decrease), a prospective single-center study in South Korea observed PIH in 26.7% with remifentanil (1 μg/kg bolus plus 0.1 μg/kg/min infusion) and in 6.7% with fentanyl (1 μg/kg). The PIH incidence in their remifentanil arm is similar to ours, whereas the rate in their fentanyl arm is much lower than ours, likely because they used a single low fentanyl dose without infusion; the consequence was poorer suppression of the intubation response, with markedly more hypertension and tachycardia after intubation in the fentanyl arm (63% and 77% vs. 0% and 10%) (Ko et al., 2013). In elderly patients (65–90 years) undergoing rapid-sequence induction with etomidate, a randomized, double-blind, three-arm trial reported PIH, which was defined as SBP <90 mmHg requiring vasopressors, in 0%, 11%, and 24% of the placebo, remifentanil 0.5 μg/kg, and 1.0 μg/kg groups, respectively (Chaumeron et al., 2020). A recent dose finding study in elderly patients defined hypotension as MAP <65 mmHg or SBP reduction >30% and recorded no events within a 3 min window after etomidate–remifentanil, which limits inference beyond the immediate period (Hao et al., 2025). Although not directly comparable, these findings align with our observation that remifentanil provides more stable hemodynamics than fentanyl during etomidate induction.

Under propofol based induction, comparative studies have yielded heterogeneous findings. Some report more hypotension with remifentanil, while others show acceptable stability depending on dose and patient risk (Manukyan et al., 2025; Hemantkumar et al., 2024; Joshi et al., 2002; Twersky et al., 2001). In cardiac surgery with midazolam induction, a clinical study found comparable induction and intubation hemodynamics between remifentanil and fentanyl, similar rates of bradycardia or hypotension, and faster onset with remifentanil (Joo et al., 2004). Taken together, these findings indicate that the relative hemodynamic effects of remifentanil versus fentanyl vary markedly with the choice of induction agent.

Subgroup analyses indicated that the association between remifentanil use and a reduced risk of PIH was consistent across all predefined clinical subgroups, including strata defined by age, hemoglobin, and albumin levels. Although slight numerical differences in effect estimates were observed, such as a reversed but non-significant association in patients with hypoalbuminemia, none of the interaction tests reached statistical significance (all *P* for interaction > 0.05), suggesting no evidence of effect heterogeneity. These findings imply that the protective effect of remifentanil is broadly applicable across various patient populations undergoing thoracoscopic esophagectomy. The observed variations in effect direction are likely due to limited sample sizes in certain subgroups rather than true differences in treatment response. Overall, the consistency of the results supports the robustness and generalizability of remifentanil’s hemodynamic benefits in this high-risk surgical cohort.

Importantly, beyond the binary incidence of hypotension, remifentanil was also associated with more stable blood pressure dynamics. SBP variability, assessed by both CV and ARV, was significantly lower in the remifentanil group. GEE analysis revealed a smoother SBP trajectory over the 15-min post-induction period, further supporting its stabilizing effect. However, these hemodynamic advantages did not translate into significant differences in clinical outcomes such as postoperative cardiovascular complications or length of hospital stay. This may reflect the limited sample size and event rates, or it may suggest that short-term hemodynamic improvements alone are insufficient to impact broader postoperative endpoints. Nonetheless, prior studies have underscored the clinical relevance of intraoperative blood pressure variability. For instance, Hirsch et al. reported a strong association between elevated blood pressure variance and early postoperative delirium in elderly patients (Hirsch et al., 2015). Other studies have suggested an association between hemodynamic instability and adverse outcomes such as impaired organ perfusion and prolonged recovery (Putowski et al., 2022). While our study did not observe such outcome differences, the reduced blood pressure variability with remifentanil may still carry physiological benefits, particularly in frail or hemodynamically labile patients. A notable adverse effect observed in this study was the higher incidence of bradycardia in the remifentanil group. This aligns with previous reports noting remifentanil’s potential to induce sinus node suppression, atrioventricular block, and vagally mediated bradycardia (Fattorini et al., 2003; Hayashi and Tanaka, 2016). In patients with high vagal tone, baseline bradycardia, or underlying conduction abnormalities, caution is warranted, especially when administering remifentanil as a single bolus or at high initial doses (Wujtewicz et al., 2016). Close monitoring and appropriate dose titration are recommended in these scenarios.

## Limitations

Nonetheless, the present study has several limitations. First, as a retrospective observational study, it is inherently susceptible to confounding bias. Although we used a DAG framework and conducted a LASSO-based sensitivity analysis to adjust for known confounders, the possibility of residual or unmeasured confounding cannot be completely excluded. Second, the study focused on intraoperative hemodynamic parameters and short-term outcomes. Long-term postoperative endpoints, such as organ dysfunction, 30-day mortality, or functional recovery, were not assessed, limiting the ability to evaluate the full clinical impact of improved hemodynamic stability. Third, all data were derived from a single center and involved a specific high-risk surgical population (patients undergoing thoracoscopic esophagectomy). This may limit the generalizability of the findings to other patient groups or surgical procedures. Fourth, although subgroup analyses revealed no significant heterogeneity, the relatively small sample sizes in some strata (e.g., patients with anemia or hypoalbuminemia) may have reduced the statistical power to detect potential effect modification.

## Conclusion

Remifentanil-based induction was associated with a significantly lower incidence of PIH and more stable hemodynamics compared to fentanyl in patients undergoing thoracoscopic esophagectomy. These effects were consistent across clinical subgroups, suggesting that remifentanil may be a more suitable induction opioid for high-risk surgical populations.

## Data Availability

The data analyzed in this study is subject to the following licenses/restrictions: It may be available for use from the corresponding authors upon reasonable request after publication. Requests to access these datasets should be directed to Baoxin Wang, wbxshgh@163.com.
