# Supplementary material for: Remifentanil reduces post-induction hypotension compared to fentanyl in thoracoscopic esophagectomy: a retrospective cohort study
Source: Front Pharmacol. 2025 Oct 9;16:1660228. doi: 10.3389/fphar.2025.1660228 (PMC12546277; doi:10.3389/fphar.2025.1660228)
Supplement: Supplementary file 1 [file Supplementaryfile1.docx]

## **Supplementary Table S1. Multivariate Logistic Regression Analysis based on** **DAG-guided Modeling**

| Variable | aOR (95% CI) | *P* value |  |
| --- | --- | --- | --- |
| Remifentanil group | 0.42 (0.25–0.73) | 0.002 |  |
| Hypertension | 2.22 (1.23–3.79) | 0.003 |  |
| Surgery time (Morning) | 0.75 (0.45–1.27) | 0.29 |  |
| BMI | | 0.98 (0.91–1.08) | 0.62 |
| Age | 1.02 (0.98–1.05) | 0.69 |  |
| Coronary Artery Disease | 1.16 (0.52–2.40) | 0.75 |  |

Multivariable logistic regression of the primary outcome (PIH) with covariates prespecified by a DAG: age, BMI, hypertension, coronary artery disease, and surgery time (morning vs afternoon). Exposure is remifentanil with fentanyl as the reference. Continuous variables are per 1-unit increase. Reported values are aOR with 95% CIs and two-sided P values.

Abbreviations: DAG, directed acyclic graph; BMI, body mass index; aOR, adjusted odds ratio; PIH, post-induction hypotension; CI, confidence interval.

**Supplementary Table S2. Multivariable logistic regression results based on LASSO-selected variable**

| Variable | aOR (95% CI) | | *P*-value |  |
| --- | --- | --- | --- | --- |
| Remifentanil Group | 0.41 (0.22–0.69) | 0.001 | |  |
| ARB/ACEI use | 3.58 (1.72–7.59) | 0.0007 | |  |
| CCB use | | 0.33 (0.15–0.69) | 0.004 | |
| Hypertension | | 2.04 (0.97–4.32) | 0.061 | |
| Neoadjuvant chemotherapy | 1.78 (0.92–3.45) | 0.085 | |  |
| Sex (Female) | 1.52 (0.75–3.02) | 0.236 | |  |
| Hb | 0.99 (0.98–1.01) | 0.325 | |  |

Multivariable logistic regression of PIH with predictors selected by LASSO (tenfold cross-validation at λ_min). The exposure is remifentanil (reference fentanyl). Continuous predictors are interpreted per one-unit increase; binary predictors are coded yes vs no (sex = female vs male). ARB/ACEI and CCB indicate preoperative oral antihypertensive use; neoadjuvant chemotherapy is coded yes vs no. Results are reported as aORs with 95% CIs and two-sided P values.

Abbreviations: ARB, angiotensin II receptor blocker; ACEI, angiotensin-converting enzyme inhibitor; CCB, calcium channel blocker; LASSO, least absolute shrinkage and selection operator; PIH, post-induction hypotension; aOR, adjusted odds ratio; CI, confidence interval, Hb, Hemoglobin.

**Supplementary Table S3.** **Incidence of PIH based on MAP definition**

| Outcome | Fentanyl group | Remifentanil group | Difference or OR (95% CI) | *P* value |
| --- | --- | --- | --- | --- |
| PIH, No (%) | 91 (59.1) | 51 (37.8) |  |  |
| Crude OR |  |  | 0.45 (0.29–0.69) | P<0.001 |
| Adjusted OR (DAG model) |  |  | 0.42 (0.26–0.68) | P=0.004 |

PIH (MAP-based) defined as minimum MAP at 0, 5, 10, or 15 minutes < 65 mmHg or ≥ 20% decrease from baseline. “Adjusted OR (DAG model)” from multivariable logistic regression with DAG-prespecified covariates (age, BMI, hypertension, coronary artery disease, surgery time [morning vs afternoon]). Odds ratios compare remifentanil with fentanyl (values < 1 favor remifentanil). Two-sided P values.

Abbreviations: PIH, post-induction hypotension; MAP, mean arterial pressure; OR, odds ratio; CI, confidence interval; DAG, directed acyclic graph.

## **Supplementary Table S4. Multivariable logistic regression for PIH (MAP definition, adjusted for covariates identified by DAG)**

| Variable | aOR (95% CI) | *P* value |  |
| --- | --- | --- | --- |
| Remifentanil group | 0.42 (0.26–0.68) | 0.004 |  |
| Hypertension | 1.34 (0.82–2.20) | 0.24 |  |
| Surgery time (AM) | 0.79 (0.49–1.28) | 0.34 |  |
| BMI | | 0.95 (0.88–1.03) | 0.26 |
| Age | 1.00 (0.98–1.03) | 0.85 |  |
| Coronary Artery Disease | 1.31 (0.63–2.73) | 0.47 |  |

Multivariable logistic regression of PIH using the MAP definition (minimum MAP at 0, 5, 10, or 15 minutes < 65 mmHg or ≥ 20% decrease from baseline). Covariates were prespecified by a directed acyclic graph (DAG): age, BMI, hypertension, coronary artery disease, and surgery time (morning vs afternoon). Exposure is remifentanil (reference fentanyl). Continuous predictors are per 1-unit increase. Results are aORs with 95% CIs; two-sided P values.

Abbreviations: PIH, post-induction hypotension; MAP, mean arterial pressure; aOR, adjusted odds ratio; CI, confidence interval; DAG, directed acyclic graph; BMI, body mass index.

**Table S5. Model diagnostics for multivariable logistic regression (DAG-guided and LASSO-selected models).**

| Diagnostic check | DAG-guided model | LASSO-selected model | Interpretation |
| --- | --- | --- | --- |
| Linearity of logit | Age *P* = 0.195, BMI *P* = 0.113 | Hb *P* = 0.412 | No violation |
| Spline sensitivity | aOR 0.42→0.40, LRT *P* = 0.082 | aOR 0.41→0.42, LRT *P* = 0.730 | Stable effect |
| Multicollinearity (VIF) | 1.02–1.08 | 1.04–2.57 | Acceptable (<3) |
| Influential observations | 11 flagged | 9 flagged | Estimates robust |
| Separation | None | None |  |
| Discrimination (AUC) | 0.673 | 0.761 | Acceptable |
| Calibration (Hos–Lemeshow) | *P*=0.84 | *P*=0.46 | Good fit |

Only continuous covariates (age, BMI, hemoglobin) were assessed for linearity of the logit using Box–Tidwell tests; categorical variables (e.g., remifentanil group, hypertension, coronary artery disease, neoadjuvant chemotherapy, sex) were not applicable. *P* > 0.05 indicates no evidence of deviation from linearity.

**Abbreviations:** aOR, adjusted odds ratio; LRT, likelihood-ratio test; VIF, variance inflation factor; AUC, area under the receiver operating characteristic curve; Hosmer–Lemeshow, Hosmer–Lemeshow goodness-of-fit test; BMI, body mass index; Hb, Hemoglobin.

**Figure S1A. LASSO (binomial): tenfold cross-validation curve**
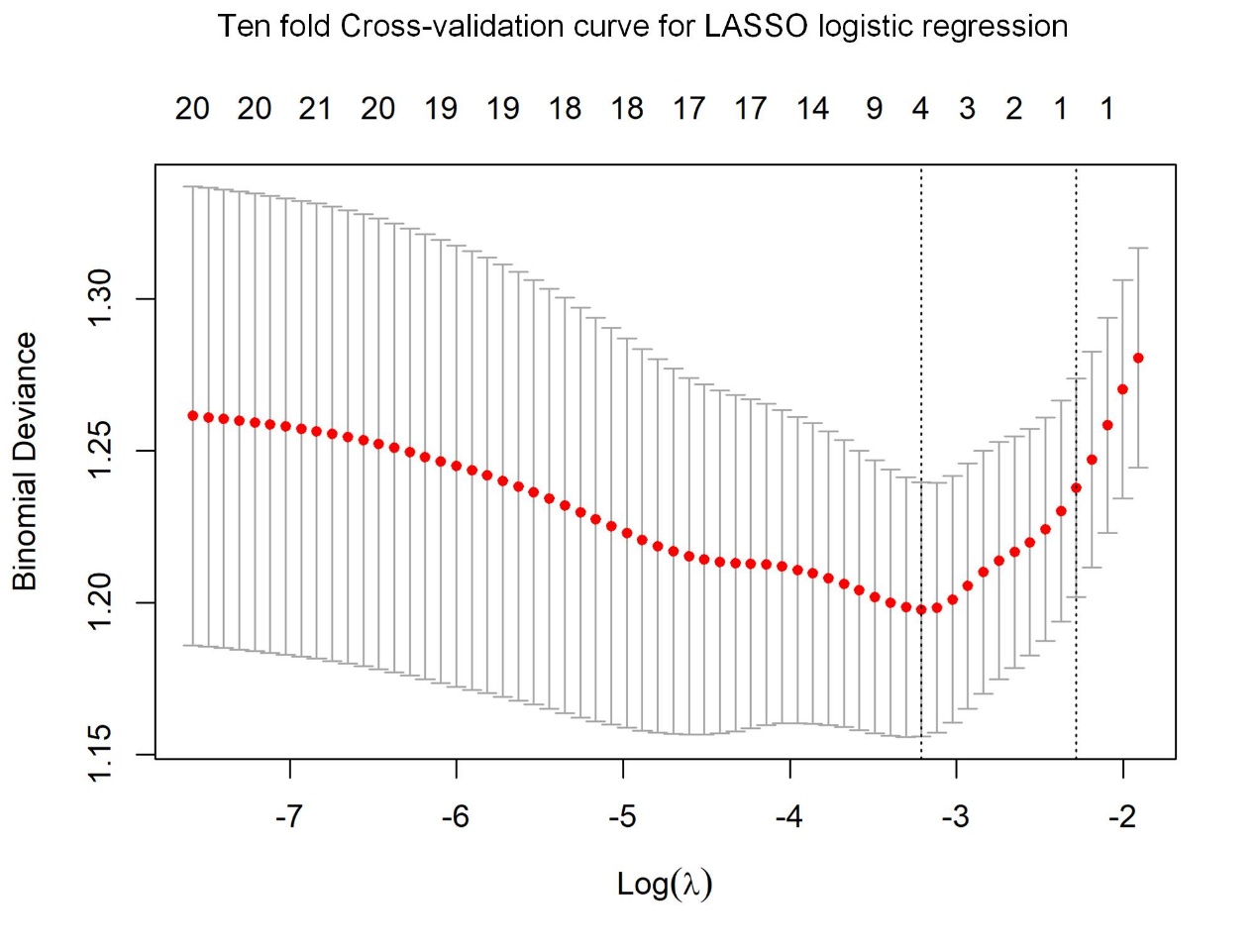


**Figure S1B. LASSO (binomial): coefficient path**
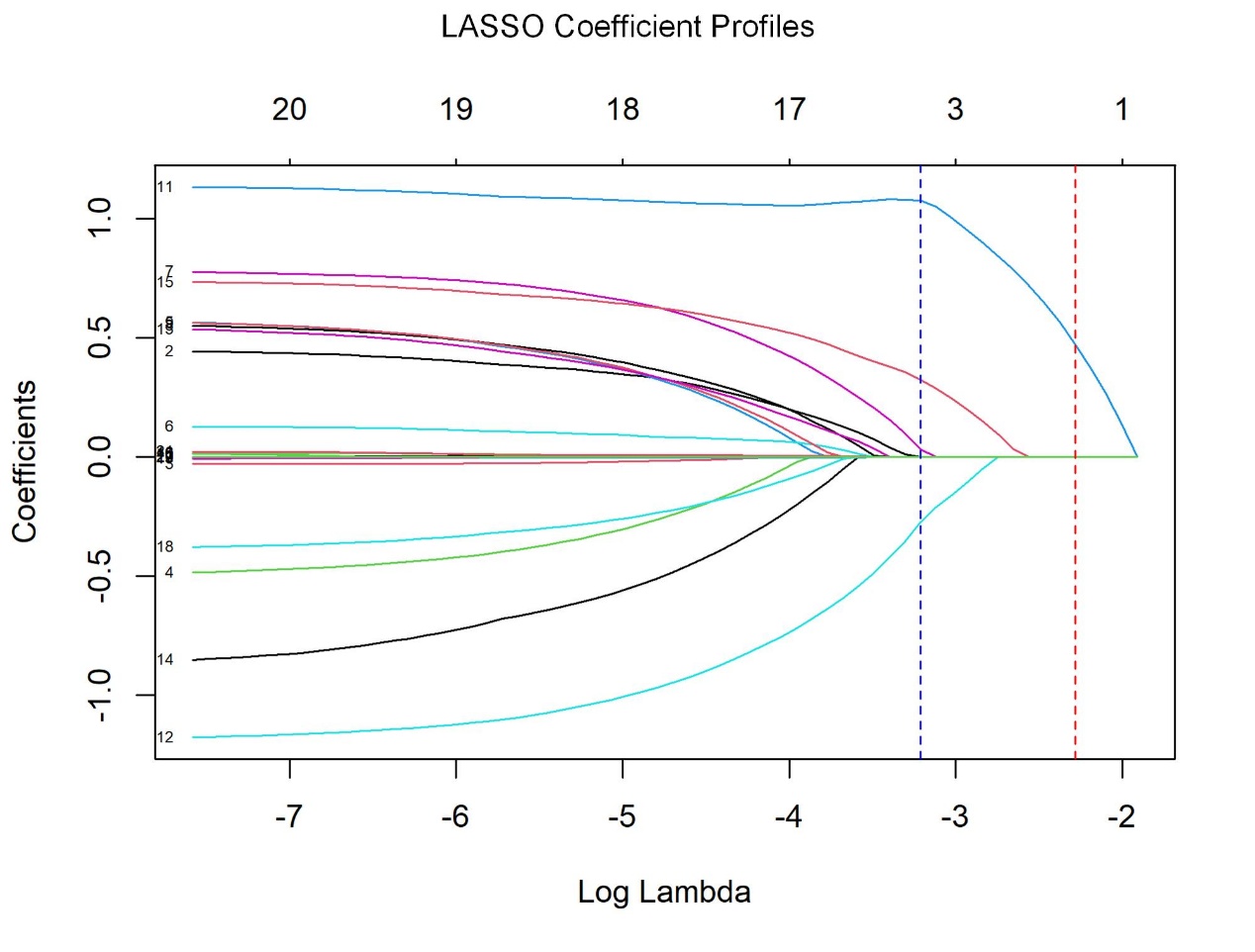


(A) LASSO (binomial): tenfold cross-validation curve. Points show mean deviance ±1 standard error (SE); vertical lines mark λ_min and λ_1se; lower is better; selection at λ_min. (B) LASSO (binomial): coefficient paths. Mean cross-validated (CV) binomial deviance (±1 SE) versus log(λ). Vertical lines mark λ_min and λ_1se. Lower values indicate better fit; selection at λ_min.

Abbreviations: LASSO, least absolute shrinkage and selection operator; CV, cross-validation; SE, standard error; λ_min, the penalty giving the minimum cross-validated deviance; λ_1se, the most regularized penalty within 1 SE of the minimum.

**Figure S2. Receiver operating characteristic (ROC) curves for prediction of post-induction hypotension (PIH).
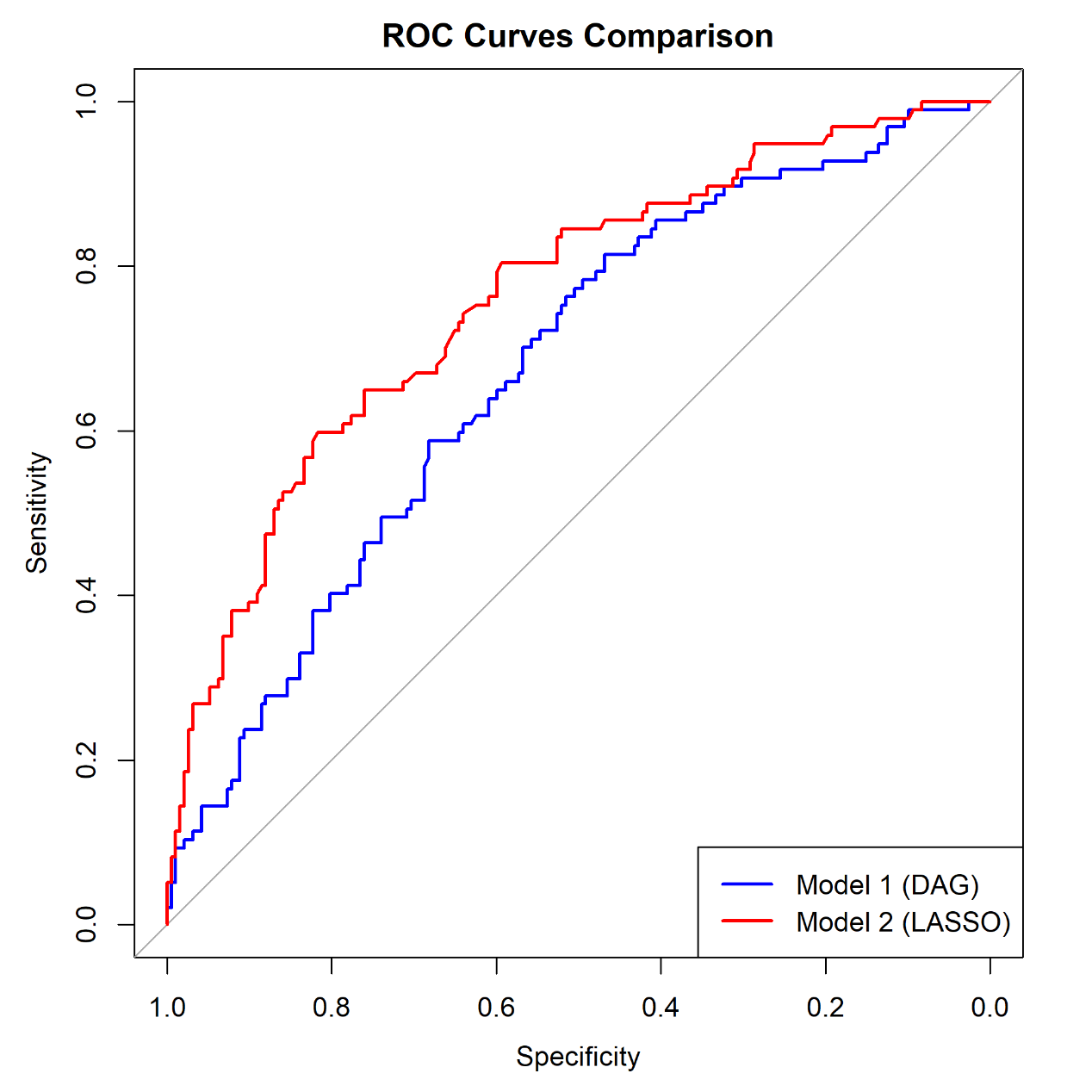
**

ROC curves for prediction of PIH. The ROC curves show the discrimination performance of the DAG-guided logistic-regression model (area under the ROC curve [AUC] = 0.673) and the LASSO-selected model (AUC = 0.761).

Abbreviations: ROC, receiver operating characteristic; AUC, area under the ROC curve; PIH, post-induction hypotension; DAG, directed acyclic graph; LASSO, least absolute shrinkage and selection operator.

**Figure S3. Calibration plots of the predicted versus observed probability of post-induction hypotension (PIH).**

**
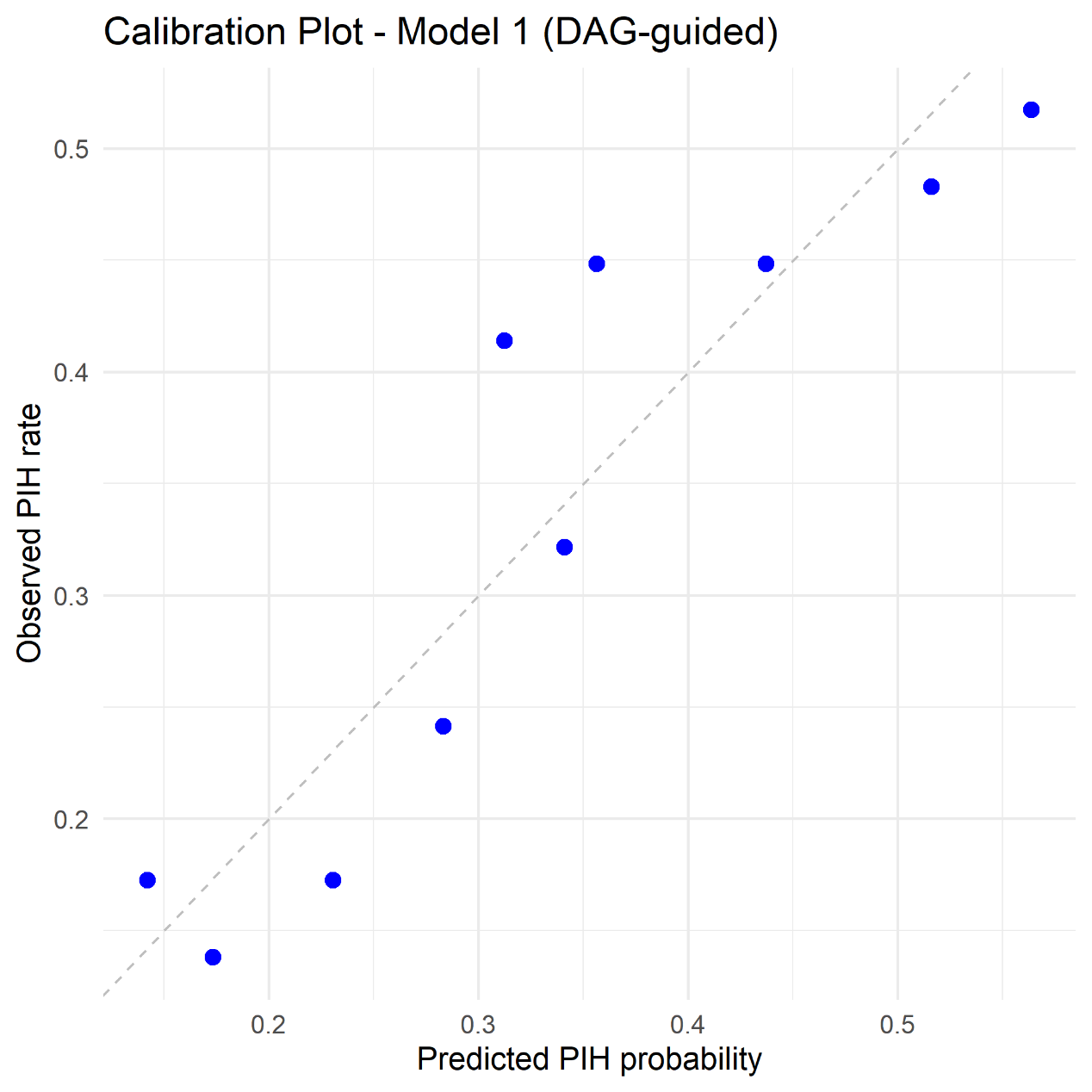
**

**
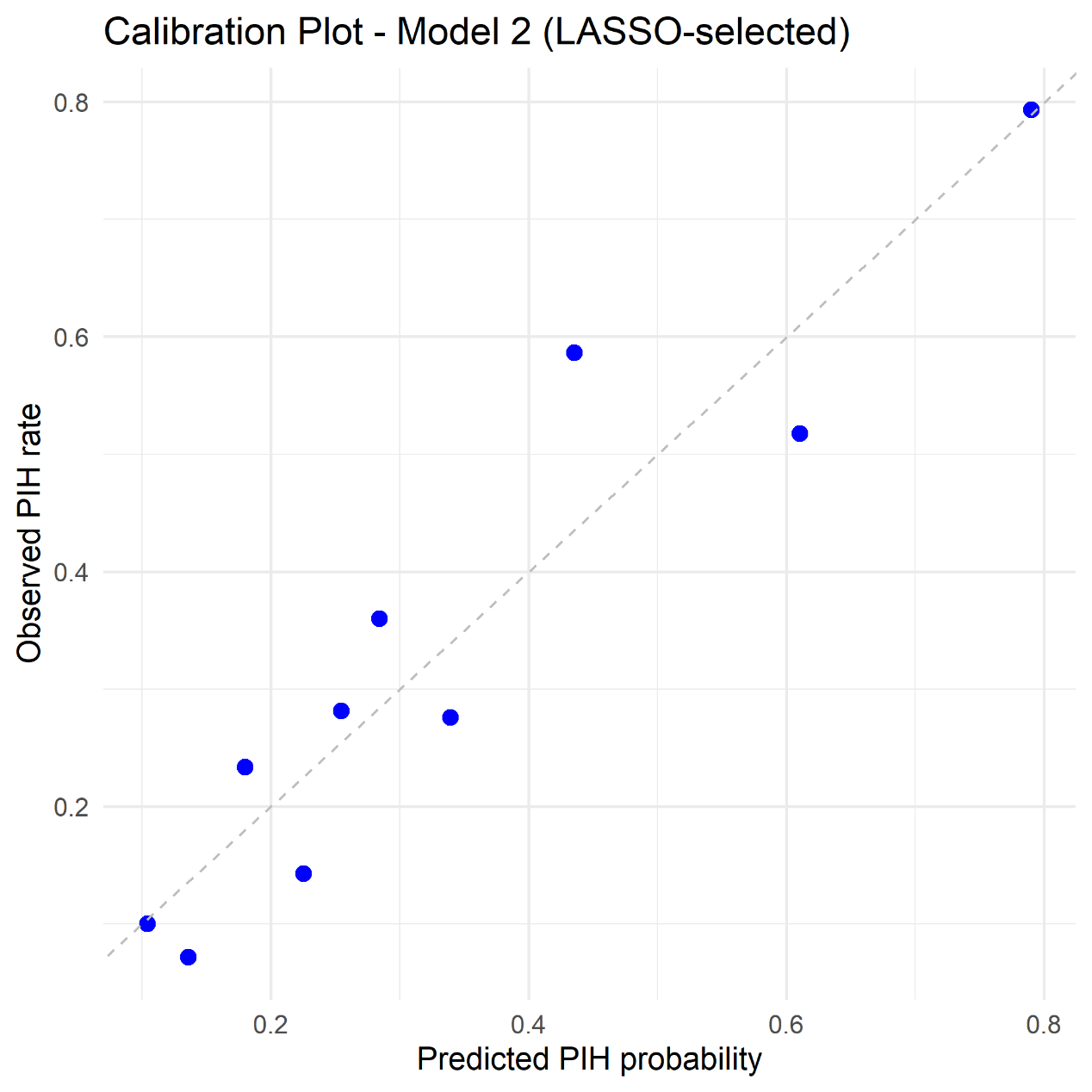
**

(A)Calibration performance is displayed for the DAG-guided model and (B) the LASSO-selected model. The dashed line represents perfect calibration.

Abbreviations: PIH, post-induction hypotension; DAG, directed acyclic graph; LASSO, least absolute shrinkage and selection operator.
