# Supplementary material for: Remifentanil reduces post-induction hypotension compared to fentanyl in thoracoscopic esophagectomy: a retrospective cohort study
Source: Front Pharmacol. 2025 Oct 9;16:1660228. doi: 10.3389/fphar.2025.1660228 (PMC12546277; doi:10.3389/fphar.2025.1660228)
Supplement: Supplementary file 2 [file Supplementaryfile2.docx]

# ============================================================

# 01) Baseline (no outcomes): summary by group

# Continuous: mean +/- SD (Welch t) if normal in both; else median [IQR] (Wilcoxon)

# Categorical: n (%) + Chi-square (Fisher if any expected cell < 5)

# Derived rows: BMI < 18.5; Albumin <= 30 g/L; Hemoglobin <= 100 g/L

# ============================================================

set.seed(20240908)

options(stringsAsFactors = FALSE)

# --- Packages (install manually if missing) ---

need <- c(

"tidyverse","janitor","writexl","broom","sandwich","lmtest",

"glmnet","geepack","emmeans","logistf"

)

miss <- need[!sapply(need, requireNamespace, quietly = TRUE)]

if (length(miss)) stop("Missing packages: ", paste(miss, collapse = ", "))

invisible(lapply(need, function(p) library(p, character.only = TRUE)))

# --- Paths / dirs ---

dir.create("outputs/01_baseline", recursive = TRUE, showWarnings = FALSE)

dir.create("outputs/02_pih", recursive = TRUE, showWarnings = FALSE)

dir.create("outputs/03_models", recursive = TRUE, showWarnings = FALSE)

dir.create("outputs/04_lasso", recursive = TRUE, showWarnings = FALSE)

dir.create("outputs/05_variab", recursive = TRUE, showWarnings = FALSE)

dir.create("outputs/06_gee", recursive = TRUE, showWarnings = FALSE)

dir.create("outputs/07_subgroup", recursive = TRUE, showWarnings = FALSE)

path_baseline <- "data/baseline.csv"

path_gee <- "data/GEE.csv"

# ============================================================

# 01) Baseline table (no outcomes included)

# - Continuous: mean±SD + Welch's t if normal in both groups; else median[IQR] + Wilcoxon

# - Categorical: n(%) + Chi-square (switch to Fisher if expected cell <5)

# - Derived rows: BMI<18.5, Albumin ≤30 g/L, Hemoglobin ≤100 g/L

# ============================================================

if (!file.exists(path_baseline)) stop("baseline.csv not found in ./data")

df <- readr::read_csv(path_baseline, show_col_types = FALSE) |> janitor::clean_names()

# Group must have two levels

if (!"group" %in% names(df)) stop("'group' column is required in baseline.csv")

if (is.numeric(df$group) || is.integer(df$group)) {

df <- df |> mutate(group = factor(group, levels = c(1,2), labels = c("Group 1","Group 2")))

} else {

if (dplyr::n_distinct(df$group) != 2) stop("'group' must have exactly two levels")

df <- df |> mutate(group = factor(group))

}

# Optional factor conversions when present

maybe_factor <- c("sex","asa","hypertension","copd","diabetes","cad",

"arb_acei","ccb","diuretic","betablocker","chemo",

"surgery_approch","surgery_approach","field","am_pm")

for (v in intersect(maybe_factor, names(df))) df[[v]] <- factor(df[[v]])

# Derived baseline categories (for table display only)

if ("bmi" %in% names(df)) {

df <- df |> mutate(bmi_lt_18_5 = factor(if_else(bmi < 18.5, "BMI < 18.5", "BMI ≥ 18.5"),

levels = c("BMI < 18.5","BMI ≥ 18.5")))

}

if ("alb" %in% names(df)) {

df <- df |> mutate(alb_le_30 = factor(if_else(alb <= 30, "Albumin ≤ 30", "Albumin > 30"),

levels = c("Albumin ≤ 30","Albumin > 30")))

}

if ("hb" %in% names(df)) {

df <- df |> mutate(hb_le_100 = factor(if_else(hb <= 100, "Hemoglobin ≤ 100", "Hemoglobin > 100"),

levels = c("Hemoglobin ≤ 100","Hemoglobin > 100")))

}

# Treat low-cardinality numerics (≤5 unique) as categorical, excluding "group"

num_cols <- names(df)[sapply(df, is.numeric)]

num_small <- num_cols[sapply(num_cols, function(x) dplyr::n_distinct(df[[x]][!is.na(df[[x]])]) <= 5)]

for (v in setdiff(num_small, "group")) df[[v]] <- factor(df[[v]])

# Lists

is_cont <- sapply(df, is.numeric)

cont_vars <- setdiff(names(df)[is_cont], "group")

is_cat <- sapply(df, is.factor) | sapply(df, is.character)

cat_vars <- setdiff(names(df)[is_cat], "group")

# Helpers

fmt_mean_sd <- function(x) sprintf("%.2f \u00B1 %.2f", mean(x, na.rm=TRUE), sd(x, na.rm=TRUE))

fmt_median_iqr <- function(x) {

q <- stats::quantile(x, c(.25,.5,.75), na.rm=TRUE)

sprintf("%.2f [%.2f\u2013%.2f]", q[2], q[1], q[3]) # median [Q1–Q3]

}

make_cont_row <- function(data, var, by) {

v <- dplyr::pull(data, {{var}}); g <- dplyr::pull(data, {{by}})

if (!is.factor(g)) g <- factor(g)

lv <- levels(g)

x1 <- v[g == lv[1]]; x1 <- x1[is.finite(x1)]

x2 <- v[g == lv[2]]; x2 <- x2[is.finite(x2)]

sh1 <- if (length(x1) >= 3) stats::shapiro.test(x1)$p.value else 0

sh2 <- if (length(x2) >= 3) stats::shapiro.test(x2)$p.value else 0

normal_both <- (sh1 > 0.05) & (sh2 > 0.05)

if (normal_both) {

tibble::tibble(

Variable = as_label(enquo(var)), Display = "mean \u00B1 SD",

`Group 1` = fmt_mean_sd(x1), `Group 2` = fmt_mean_sd(x2), Overall = fmt_mean_sd(v),

`P value` = sprintf("%.3f", stats::t.test(x1, x2, var.equal = FALSE)$p.value)

)

} else {

tibble::tibble(

Variable = as_label(enquo(var)), Display = "median [IQR]",

`Group 1` = fmt_median_iqr(x1), `Group 2` = fmt_median_iqr(x2), Overall = fmt_median_iqr(v),

`P value` = sprintf("%.3f", stats::wilcox.test(x1, x2)$p.value)

)

}

}

make_cat_block <- function(data, var, by) {

tab <- data |> dplyr::count({{by}}, {{var}}) |> dplyr::group_by({{by}}) |>

dplyr::mutate(pct = n/sum(n)) |> dplyr::ungroup()

overall <- data |> dplyr::count({{var}}) |>

dplyr::mutate({{by}} := factor("Overall",

levels = c(levels(data[[as_label(enquo(by))]]), "Overall"))) |>

dplyr::group_by({{by}}) |> dplyr::mutate(pct = n/sum(n)) |> dplyr::ungroup()

mat <- data |> dplyr::count({{by}}, {{var}}) |>

tidyr::pivot_wider(names_from = {{var}}, values_from = n, values_fill = 0) |>

dplyr::select(-{{by}}) |> as.matrix()

pval <- tryCatch({

cs <- suppressWarnings(stats::chisq.test(mat, correct = FALSE))

if (any(cs$expected < 5)) stats::fisher.test(mat)$p.value else cs$p.value

}, error = function(e) stats::fisher.test(mat)$p.value)

if (nrow(mat) < 2 || ncol(mat) < 2) pval <- NA_real_

tab |> dplyr::rename(Level = {{var}}) |>

dplyr::mutate(`n(%)` = sprintf("%d (%.1f%%)", n, pct*100)) |>

dplyr::select({{by}}, Level, `n(%)`) |>

tidyr::pivot_wider(names_from = {{by}}, values_from = `n(%)`) |>

dplyr::left_join(

overall |> dplyr::rename(Level = {{var}}) |>

dplyr::transmute(Level, Overall = sprintf("%d (%.1f%%)", n, pct*100)),

by = "Level"

) |>

dplyr::mutate(Variable = as_label(enquo(var))) |>

dplyr::relocate(Variable, Level) |>

dplyr::mutate(`P value` = ifelse(dplyr::row_number() == 1,

ifelse(is.na(pval), "", sprintf("%.3f", pval)), ""))

}

tab_cont <- purrr::map_df(cont_vars, ~ make_cont_row(df, all_of(.x), group))

tab_cat <- purrr::map_df(cat_vars, ~ make_cat_block(df, all_of(.x), group))

writexl::write_xlsx(list("Table1_Continuous" = tab_cont, "Table1_Categorical" = tab_cat),

path = "outputs/01_baseline/Table1_baseline.xlsx")

print(tab_cont, n = nrow(tab_cont))

print(tab_cat, n = nrow(tab_cat))

message("[01] Baseline table saved to outputs/01_baseline/Table1_baseline.xlsx")

# ============================================================

# 02) PIH incidence (SBP/MAP) with hemodynamic QC

# Definitions:

# PIH (SBP): min(SBP_0/5/10/15) < 90 OR >= 30% drop from baseline

# PIH (MAP): min(MAP_0/5/10/15) < 65 OR >= 20% drop from baseline

# ============================================================

if (!file.exists(path_gee)) stop("GEE.csv not found in ./data")

dat <- readr::read_csv(path_gee, show_col_types = FALSE) |> janitor::clean_names()

# Exposure coding (Fentanyl = reference)

if (!is.factor(dat$group)) {

dat <- dat |>

dplyr::mutate(

group = dplyr::case_when(

group %in% c("R","r","Remifentanil","remifentanil",1,"1") ~ "Remifentanil",

TRUE ~ "Fentanyl"

),

group = factor(group, levels = c("Fentanyl","Remifentanil"))

)

}

# ---------- Hemodynamic QC (drop implausible values -> NA) ----------

qc_params <- list(

sbp_lo = 40, sbp_hi = 300,

map_lo = 30, map_hi = 200,

hr_lo = 20, hr_hi = 220

)

qc_clip_vec <- function(x, lo, hi) {

x <- as.numeric(x)

x[!is.finite(x)] <- NA_real_

x[x <= 0] <- NA_real_ # 0/negative -> invalid

x[x < lo | x > hi] <- NA_real_ # outside physiologic bounds -> NA

x

}

dat_raw <- dat # keep an untouched copy for comparison

dat_qc <- dat

# Candidate columns in wide/long structures

hemo_cols <- intersect(

names(dat_qc),

c("sbp_base","map_base","hr_base",

"sbp_0","sbp_5","sbp_10","sbp_15",

"map_0","map_5","map_10","map_15",

"sbp","map","hr")

)

for (nm in hemo_cols) {

if (grepl("^sbp(_|$)", nm)) dat_qc[[nm]] <- qc_clip_vec(dat_qc[[nm]], qc_params$sbp_lo, qc_params$sbp_hi)

if (grepl("^map(_|$)", nm)) dat_qc[[nm]] <- qc_clip_vec(dat_qc[[nm]], qc_params$map_lo, qc_params$map_hi)

if (grepl("^hr(_|$)", nm)) dat_qc[[nm]] <- qc_clip_vec(dat_qc[[nm]], qc_params$hr_lo, qc_params$hr_hi)

}

# If both SBP and MAP are present at a time point and MAP > SBP, set MAP to NA

times <- c("base","0","5","10","15")

for (t in times) {

sb <- paste0("sbp_", t); mp <- paste0("map_", t)

if (t == "base") { sb <- "sbp_base"; mp <- "map_base" }

if (all(c(sb, mp) %in% names(dat_qc))) {

idx <- which(!is.na(dat_qc[[mp]]) & !is.na(dat_qc[[sb]]) & dat_qc[[mp]] > dat_qc[[sb]])

if (length(idx)) dat_qc[[mp]][idx] <- NA_real_

}

}

# QC summary: how many values were dropped to NA per column

dir.create("outputs/02_pih", recursive = TRUE, showWarnings = FALSE)

qc_count <- function(raw, qc, vars) {

tibble::tibble(

variable = vars,

n_pre = sapply(vars, function(v) sum(!is.na(raw[[v]]))),

n_dropped = sapply(vars, function(v) sum(!is.na(raw[[v]]) & is.na(qc[[v]]))),

pct_dropped = round(100 * n_dropped / pmax(n_pre, 1), 1)

)

}

if (length(hemo_cols)) {

qc_summary <- qc_count(dat_raw, dat_qc, hemo_cols)

readr::write_csv(qc_summary, "outputs/02_pih/qc_summary.csv")

}

# ---------- Build PIH flags from wide SBP/MAP columns: raw vs QC ----------

make_pih_flags <- function(D) {

need_sbp <- c("sbp_base","sbp_0","sbp_5","sbp_10","sbp_15")

need_map <- c("map_base","map_0","map_5","map_10","map_15")

if (!("pih_sbp" %in% names(D)) && all(need_sbp %in% names(D))) {

D <- D |>

dplyr::mutate(

min_sbp = pmin(sbp_0, sbp_5, sbp_10, sbp_15, na.rm = TRUE),

# if all four SBP timepoints are missing -> min_sbp := NA

min_sbp = dplyr::if_else(

rowSums(is.na(dplyr::select(D, sbp_0, sbp_5, sbp_10, sbp_15))) == 4,

NA_real_, min_sbp

),

sbp_drop_pct = (sbp_base - min_sbp) / sbp_base,

pih_sbp = as.integer((min_sbp < 90) | (sbp_drop_pct >= 0.30))

)

}

if (!("pih_map" %in% names(D)) && all(need_map %in% names(D))) {

D <- D |>

dplyr::mutate(

min_map = pmin(map_0, map_5, map_10, map_15, na.rm = TRUE),

# if all four MAP timepoints are missing -> min_map := NA

min_map = dplyr::if_else(

rowSums(is.na(dplyr::select(D, map_0, map_5, map_10, map_15))) == 4,

NA_real_, min_map

),

map_drop_pct = (map_base - min_map) / map_base,

pih_map = as.integer((min_map < 65) | (map_drop_pct >= 0.20))

)

}

D

}

dat_raw <- make_pih_flags(dat_raw)

dat_qc <- make_pih_flags(dat_qc)

prop_ci <- function(x, n) {

pt <- stats::prop.test(x, n)

tibble::tibble(n = n, events = x, prop = as.numeric(pt$estimate),

ci_low = unname(pt$conf.int[1]), ci_high = unname(pt$conf.int[2]))

}

inc_tbl <- function(D, which = c("SBP","MAP")) {

out <- list()

if ("SBP" %in% which && "pih_sbp" %in% names(D)) {

a <- D |>

dplyr::group_by(group) |>

dplyr::summarise(n_nonmiss = sum(!is.na(pih_sbp)),

events = sum(pih_sbp == 1, na.rm = TRUE), .groups = "drop") |>

dplyr::rowwise() |>

dplyr::mutate(tmp = list(prop_ci(events, n_nonmiss))) |>

tidyr::unnest_wider(tmp) |>

dplyr::mutate(metric = "PIH (SBP)") |>

dplyr::relocate(metric)

out$sbp <- a

}

if ("MAP" %in% which && "pih_map" %in% names(D)) {

b <- D |>

dplyr::group_by(group) |>

dplyr::summarise(n_nonmiss = sum(!is.na(pih_map)),

events = sum(pih_map == 1, na.rm = TRUE), .groups = "drop") |>

dplyr::rowwise() |>

dplyr::mutate(tmp = list(prop_ci(events, n_nonmiss))) |>

tidyr::unnest_wider(tmp) |>

dplyr::mutate(metric = "PIH (MAP)") |>

dplyr::relocate(metric)

out$map <- b

}

dplyr::bind_rows(out)

}

inc_raw <- inc_tbl(dat_raw, c("SBP","MAP"))

inc_qc <- inc_tbl(dat_qc, c("SBP","MAP"))

readr::write_csv(inc_raw, "outputs/02_pih/pih_incidence_by_group_raw.csv")

readr::write_csv(inc_qc, "outputs/02_pih/pih_incidence_by_group_qc.csv")

inc_cmp <- inc_qc |>

dplyr::rename(n_nonmiss_qc = n_nonmiss, events_qc = events,

prop_qc = prop, ci_low_qc = ci_low, ci_high_qc = ci_high) |>

dplyr::left_join(

inc_raw |>

dplyr::rename(n_nonmiss_raw = n_nonmiss, events_raw = events,

prop_raw = prop, ci_low_raw = ci_low, ci_high_raw = ci_high),

by = c("metric","group")

)

readr::write_csv(inc_cmp, "outputs/02_pih/pih_incidence_compare.csv")

message("[02] Incidence (raw & QC) saved to outputs/02_pih/")

# From here on, analyses use QC data only.

dat <- dat_qc

# ============================================================

# 03) Multivariable logistic models (DAG covariates; QC data)

# ============================================================

dag_covars <- c("age","bmi","hbp","cad","am_pm")

tidy_coeftest <- function(ct) {

out <- as.data.frame(ct) |> tibble::rownames_to_column("term")

names(out) <- c("term","estimate","std.error","statistic","p.value")

tibble::as_tibble(out)

}

# SBP model

df_sbp <- dat |> dplyr::select(dplyr::all_of(c("pih_sbp","group", dag_covars))) |> tidyr::drop_na()

fit_sbp <- stats::glm(pih_sbp ~ group + age + bmi + hbp + cad + am_pm, data = df_sbp, family = stats::binomial())

tab_sbp <- broom::tidy(fit_sbp, conf.int = TRUE, conf.level = 0.95, exponentiate = TRUE) |>

dplyr::mutate(dplyr::across(c(estimate, conf.low, conf.high), ~ round(.x, 3)),

p.value = signif(p.value, 3))

readr::write_csv(tab_sbp, "outputs/03_models/logit_sbp_or_ci.csv")

rob_sbp <- lmtest::coeftest(fit_sbp, vcov = sandwich::vcovHC(fit_sbp, type = "HC0"))

tidy_coeftest(rob_sbp) |> readr::write_csv("outputs/03_models/logit_sbp_robust_se.csv")

# MAP model

df_map <- dat |> dplyr::select(dplyr::all_of(c("pih_map","group", dag_covars))) |> tidyr::drop_na()

fit_map <- stats::glm(pih_map ~ group + age + bmi + hbp + cad + am_pm, data = df_map, family = stats::binomial())

tab_map <- broom::tidy(fit_map, conf.int = TRUE, conf.level = 0.95, exponentiate = TRUE) |>

dplyr::mutate(dplyr::across(c(estimate, conf.low, conf.high), ~ round(.x, 3)),

p.value = signif(p.value, 3))

readr::write_csv(tab_map, "outputs/03_models/logit_map_or_ci.csv")

rob_map <- lmtest::coeftest(fit_map, vcov = sandwich::vcovHC(fit_map, type = "HC0"))

tidy_coeftest(rob_map) |> readr::write_csv("outputs/03_models/logit_map_robust_se.csv")

message("[03] Logistic models (QC) saved.")

# ============================================================

# 04) LASSO (primary SBP PIH, QC) + refit

# ============================================================

if (!("arb_acei" %in% names(dat))) {

if (all(c("arb","acei") %in% names(dat))) {

dat <- dat |> dplyr::mutate(arb_acei = as.integer((arb %in% c(1,"1",TRUE)) | (acei %in% c(1,"1",TRUE))))

} else if ("arb" %in% names(dat)) {

dat <- dat |> dplyr::mutate(arb_acei = as.integer(arb %in% c(1,"1",TRUE)))

} else if ("acei" %in% names(dat)) {

dat <- dat |> dplyr::mutate(arb_acei = as.integer(acei %in% c(1,"1",TRUE)))

}

}

candidates <- intersect(

c("group","age","sex","bmi","hbp","cad","am_pm","chemo","arb_acei","ccb","alb","hb","sbp_base","map_base"),

names(dat)

)

df_lasso <- dat |> dplyr::select(dplyr::all_of(c("pih_sbp", candidates))) |> tidyr::drop_na()

if (nrow(df_lasso) < 10) stop("Too few complete cases for LASSO.")

x <- model.matrix(pih_sbp ~ ., data = df_lasso)[, -1]

y <- df_lasso$pih_sbp

set.seed(20240908)

cvfit <- glmnet::cv.glmnet(x, y, family = "binomial", alpha = 1, nfolds = 10, standardize = TRUE)

lambda_min <- cvfit$lambda.min; lambda_1se <- cvfit$lambda.1se

coef_min <- coef(cvfit, s = lambda_min)

sel_terms <- rownames(coef_min)[as.numeric(coef_min) != 0]

sel_terms <- setdiff(sel_terms, "(Intercept)")

orig_cols <- intersect(sel_terms, colnames(df_lasso))

if (!"group" %in% orig_cols && "group" %in% colnames(df_lasso)) orig_cols <- c("group", orig_cols)

if (!length(orig_cols)) orig_cols <- "group"

form_lasso <- as.formula(paste("pih_sbp ~", paste(orig_cols, collapse = " + ")))

fit_lasso <- stats::glm(form_lasso, data = df_lasso, family = stats::binomial())

tab_lasso <- broom::tidy(fit_lasso, conf.int = TRUE, conf.level = 0.95, exponentiate = TRUE) |>

dplyr::mutate(dplyr::across(c(estimate, conf.low, conf.high), ~ round(.x, 3)), p.value = signif(p.value, 3))

dir.create("outputs/04_lasso", recursive = TRUE, showWarnings = FALSE)

readr::write_csv(tibble::tibble(lambda_min = lambda_min, lambda_1se = lambda_1se), "outputs/04_lasso/lasso_lambdas.csv")

readr::write_csv(tibble::tibble(selected_design_terms = sel_terms), "outputs/04_lasso/lasso_selected_terms_design_matrix.csv")

readr::write_csv(tibble::tibble(selected_original_columns = orig_cols), "outputs/04_lasso/lasso_selected_original_columns.csv")

readr::write_csv(tab_lasso, "outputs/04_lasso/lasso_glm_or_ci.csv")

png("outputs/04_lasso/lasso_cv_curve.png", width = 1400, height = 900, res = 300); plot(cvfit); dev.off()

png("outputs/04_lasso/lasso_coef_path.png", width = 1400, height = 900, res = 300)

plot(glmnet::glmnet(x, y, family = "binomial", alpha = 1, standardize = TRUE)); dev.off()

message("[04] LASSO results (QC) saved.")

# ============================================================

# 05) SBP variability 0-15 min (ARV & CV) + group tests

# ============================================================

need_sbp4 <- c("sbp_0","sbp_5","sbp_10","sbp_15")

if (!all(need_sbp4 %in% names(dat))) {

warning("[05] Variability skipped: sbp_0/5/10/15 not all present.")

} else {

row_arv <- function(v) { v <- v[is.finite(v)]; if (length(v) < 2) return(NA_real_); mean(abs(diff(v))) }

sbp_mat <- dat |> dplyr::select(dplyr::all_of(need_sbp4))

sbp_mean <- apply(sbp_mat, 1, function(z) mean(z, na.rm = TRUE))

sbp_sd <- apply(sbp_mat, 1, function(z) sd(z, na.rm = TRUE))

sbp_arv <- apply(sbp_mat, 1, row_arv)

sbp_cv <- 100 * (sbp_sd / sbp_mean)

dat_var <- dat |> dplyr::mutate(sbp_arv_0_15 = sbp_arv, sbp_cv_0_15 = sbp_cv)

fmt_mean_sd2 <- function(x) sprintf("%.2f +/- %.2f", mean(x, na.rm=TRUE), sd(x, na.rm=TRUE))

fmt_med_iqr2 <- function(x) { q <- stats::quantile(x, c(.25,.5,.75), na.rm=TRUE); sprintf("%.2f [%.2f, %.2f]", q[2], q[1], q[3]) }

summ_by_group <- dat_var |>

dplyr::group_by(group) |>

dplyr::summarise(n = dplyr::n(),

ARV_mean_SD = fmt_mean_sd2(sbp_arv_0_15),

ARV_median_IQR = fmt_med_iqr2(sbp_arv_0_15),

CV_mean_SD = fmt_mean_sd2(sbp_cv_0_15),

CV_median_IQR = fmt_med_iqr2(sbp_cv_0_15),

.groups = "drop")

is_normal <- function(x) { x <- x[is.finite(x)]; if (length(x) < 3) return(FALSE); stats::shapiro.test(x)$p.value > 0.05 }

x_arv_F <- dat_var$sbp_arv_0_15[dat_var$group == "Fentanyl"]

x_arv_R <- dat_var$sbp_arv_0_15[dat_var$group == "Remifentanil"]

x_cv_F <- dat_var$sbp_cv_0_15[ dat_var$group == "Fentanyl"]

x_cv_R <- dat_var$sbp_cv_0_15[ dat_var$group == "Remifentanil"]

arv_test <- if (is_normal(x_arv_F) & is_normal(x_arv_R)) {

list(metric = "SBP-ARV (0-15 min)", test = "Welch t-test", p = stats::t.test(x_arv_F, x_arv_R, var.equal = FALSE)$p.value)

} else {

list(metric = "SBP-ARV (0-15 min)", test = "Wilcoxon rank-sum", p = stats::wilcox.test(x_arv_F, x_arv_R)$p.value)

}

cv_test <- if (is_normal(x_cv_F) & is_normal(x_cv_R)) {

list(metric = "SBP-CV (0-15 min)", test = "Welch t-test", p = stats::t.test(x_cv_F, x_cv_R, var.equal = FALSE)$p.value)

} else {

list(metric = "SBP-CV (0-15 min)", test = "Wilcoxon rank-sum", p = stats::wilcox.test(x_cv_F, x_cv_R)$p.value)

}

pvals_tbl <- tibble::tibble(metric = c(arv_test$metric, cv_test$metric),

test = c(arv_test$test, cv_test$test),

p_value = signif(c(arv_test$p, cv_test$p), 3))

dir.create("outputs/05_variab", recursive = TRUE, showWarnings = FALSE)

readr::write_csv(dat_var |> dplyr::select(group, sbp_arv_0_15, sbp_cv_0_15), "outputs/05_variab/sbp_variability_rowwise.csv")

readr::write_csv(summ_by_group, "outputs/05_variab/sbp_variability_group_summary.csv")

readr::write_csv(pvals_tbl, "outputs/05_variab/sbp_variability_pvalues.csv")

message("[05] Variability results (QC) saved.")

}

# ============================================================

# 06) GEE: SBP & HR trajectories at 0/5/10/15 (QC data)

# ============================================================

req <- c("patient_id","time","sbp","hr","age","bmi","hbp","am_pm","cad","group")

if (!all(req %in% names(dat))) {

message("[06] GEE skipped (required long-format columns not all present).")

} else {

dgee <- dat |>

dplyr::mutate(time = factor(time, levels = c(0,5,10,15), ordered = TRUE),

hbp = factor(hbp), am_pm = factor(am_pm), cad = factor(cad)) |>

tidyr::drop_na(patient_id, time, sbp, hr, group, hbp, am_pm, bmi, age, cad)

dir.create("outputs/06_gee", recursive = TRUE, showWarnings = FALSE)

gee_sbp <- geepack::geeglm(sbp ~ group * time + hbp + am_pm + bmi + age + cad,

id = patient_id, data = dgee, family = gaussian(), corstr = "ar1")

emm_sbp <- emmeans::emmeans(gee_sbp, ~ group | time)

cmp_sbp <- emmeans::contrast(emm_sbp, method = "revpairwise")

res_tbl_sbp <- broom::tidy(cmp_sbp) |>

dplyr::transmute(time, contrast,

estimate = round(estimate, 2),

SE = round(std.error, 2),

`95% CI low` = round(estimate - 1.96*std.error, 2),

`95% CI high` = round(estimate + 1.96*std.error, 2),

p.value = signif(p.value, 3))

readr::write_csv(res_tbl_sbp, "outputs/06_gee/gee_sbp_diffs.csv")

gee_hr <- geepack::geeglm(hr ~ group * time + hbp + am_pm + bmi + age + cad,

id = patient_id, data = dgee, family = gaussian(), corstr = "ar1")

emm_hr <- emmeans::emmeans(gee_hr, ~ group | time)

cmp_hr <- emmeans::contrast(emm_hr, method = "revpairwise")

res_tbl_hr <- broom::tidy(cmp_hr) |>

dplyr::transmute(time, contrast,

estimate = round(estimate, 2),

SE = round(std.error, 2),

`95% CI low` = round(estimate - 1.96*std.error, 2),

`95% CI high` = round(estimate + 1.96*std.error, 2),

p.value = signif(p.value, 3))

readr::write_csv(res_tbl_hr, "outputs/06_gee/gee_hr_diffs.csv")

# Optional plotting omitted (keep your plotting code if needed)

message("[06] GEE results (QC) saved.")

}

# ============================================================

# 07) Subgroup analysis (SBP only; QC data)

# ============================================================

stopifnot("pih_sbp" %in% names(dat))

to_yesno <- function(x) {

y <- ifelse(is.na(x), NA_character_, ifelse(x %in% c(1,"1",TRUE,"TRUE","Yes","yes","Y","y"), "Yes", "No"))

factor(y, levels = c("Yes","No"))

}

if ("age" %in% names(dat)) dat <- dat |> dplyr::mutate(age_gt70 = factor(dplyr::if_else(age > 70, ">70", "<=70"),

levels = c(">70","<=70")))

if ("hbp" %in% names(dat)) dat$hbp_fac <- to_yesno(dat$hbp)

if ("arb_acei" %in% names(dat)) dat$arb_acei_use <- to_yesno(dat$arb_acei)

if ("ccb" %in% names(dat)) dat$ccb_use <- to_yesno(dat$ccb)

if ("chemo" %in% names(dat)) dat$chemo_use <- to_yesno(dat$chemo)

if ("alb" %in% names(dat)) dat$alb_grp <- factor(dplyr::if_else(dat$alb <= 35, "<=35 g/L", ">35 g/L"),

levels = c("<=35 g/L",">35 g/L"))

if ("hb" %in% names(dat)) dat$hb_grp <- factor(dplyr::if_else(dat$hb <= 100,"<=100 g/L",">100 g/L"),

levels = c("<=100 g/L",">100 g/L"))

subgroups <- c("age_gt70","hbp_fac","arb_acei_use","ccb_use","chemo_use","alb_grp","hb_grp")

subgroups <- subgroups[subgroups %in% names(dat)]

is_sparse <- function(df, subgroup, outcome = "pih_sbp") {

levs <- levels(df[[subgroup]])

for (lv in levs) {

sub <- df[df[[subgroup]] == lv, , drop = FALSE]

if (nrow(sub) < 15) return(TRUE)

tab <- table(sub$group, sub[[outcome]])

if (any(tab == 0)) return(TRUE)

}

FALSE

}

rhs_from <- function(vars) if (length(vars)) paste(vars, collapse = " + ") else "1"

or_by_level_mle <- function(fit, subgroup, levels_vec) {

cf <- coef(fit); V <- sandwich::vcovHC(fit, type = "HC0")

grp2 <- grep("^group", names(cf), value = TRUE)[1]

purrr::map_dfr(seq_along(levels_vec), function(i){

lev <- as.character(levels_vec[i])

if (i == 1) {

L <- rep(0, length(cf)); names(L) <- names(cf); L[grp2] <- 1

} else {

int_candidates <- c(paste0(grp2, ":", subgroup, lev), paste0(subgroup, lev, ":", grp2))

int_name <- int_candidates[int_candidates %in% names(cf)][1]

L <- rep(0, length(cf)); names(L) <- names(cf); L[grp2] <- 1

if (!is.na(int_name)) L[int_name] <- 1

}

logOR <- sum(L*cf); SE <- sqrt(as.numeric(t(L) %*% V %*% L))

z <- logOR/SE; p <- 2*pnorm(-abs(z))

tibble::tibble(level = lev, OR = exp(logOR),

CI_low = exp(logOR - 1.96*SE), CI_high = exp(logOR + 1.96*SE), p = p)

})

}

counts_display <- function(df, subgroup) {

tot <- df |> dplyr::count(group, name = "N_group")

df |> dplyr::count(group, !!rlang::sym(subgroup), name = "n_level") |>

dplyr::left_join(tot, by = "group") |>

dplyr::mutate(pct = 100 * n_level / N_group,

display = sprintf("%d (%.1f%%)", n_level, pct)) |>

dplyr::select(group, level = !!rlang::sym(subgroup), display) |>

tidyr::pivot_wider(names_from = group, values_from = display, values_fill = "")

}

fit_firth_level <- function(df, outcome, covars) {

fml <- as.formula(paste(outcome, "~", paste(c("group", covars), collapse = " + ")))

fit <- tryCatch(logistf::logistf(fml, data = df), error = function(e) NULL)

if (is.null(fit)) return(tibble::tibble(OR = NA_real_, CI_low = NA_real_, CI_high = NA_real_, p = NA_real_))

param <- grep("^group", names(fit$coefficients), value = TRUE)[1]

or <- unname(exp(fit$coefficients[param]))

ci <- tryCatch(exp(confint(fit, parm = param)), error = function(e) c(NA_real_, NA_real_))

p <- unname(fit$prob[param])

tibble::tibble(OR = or, CI_low = ci[1], CI_high = ci[2], p = p)

}

run_one_subgroup <- function(data, subgroup, dag_covars, outcome = "pih_sbp") {

keep <- unique(c(outcome, "group", subgroup, dag_covars))

df <- data |> dplyr::select(dplyr::all_of(keep)) |> tidyr::drop_na()

if (nrow(df) < 10 || dplyr::n_distinct(df$group) < 2 || dplyr::n_distinct(df[[subgroup]]) < 2) return(tibble::tibble())

if (!is.factor(df[[subgroup]])) df[[subgroup]] <- factor(df[[subgroup]])

levs <- levels(df[[subgroup]]); dag_use <- setdiff(dag_covars, subgroup)

if (!is_sparse(df, subgroup, outcome)) {

f_base <- as.formula(paste(outcome, "~", rhs_from(c("group", subgroup, dag_use))))

f_int <- as.formula(paste(outcome, "~", rhs_from(c(paste0("group*", subgroup), dag_use))))

fit_base <- stats::glm(f_base, data = df, family = stats::binomial())

fit_int <- stats::glm(f_int, data = df, family = stats::binomial())

p_inter <- tryCatch(tail(anova(fit_base, fit_int, test = "LRT")$`Pr(>Chi)`, 1), error = function(e) NA_real_)

or_tbl <- or_by_level_mle(fit_int, subgroup, levs) |>

dplyr::mutate(OR = round(OR, 3), CI_low = round(CI_low, 3), CI_high = round(CI_high, 3),

`P value` = signif(p, 3)) |>

dplyr::select(level, OR, CI_low, CI_high, `P value`)

cnt <- counts_display(df, subgroup)

out <- cnt |> dplyr::right_join(or_tbl, by = "level") |>

dplyr::mutate(Subgroup = subgroup,

`P for interaction` = ifelse(is.na(p_inter), NA, signif(p_inter, 3)),

Method = "Interaction (MLE)") |>

dplyr::relocate(Subgroup, level, OR, CI_low, CI_high, `P value`, `P for interaction`, Method)

return(out)

} else {

rows <- purrr::map_dfr(levs, function(lv){

df_lv <- df |> dplyr::filter(.data[[subgroup]] == lv)

est <- fit_firth_level(df_lv, outcome, dag_use)

cnt <- counts_display(df_lv, subgroup) |> dplyr::mutate(level = lv)

dplyr::left_join(cnt, est, by = "level") |>

dplyr::mutate(Subgroup = subgroup, `P for interaction` = NA, Method = "Firth (per level)")

})

rows |>

dplyr::mutate(OR = round(OR, 3), CI_low = round(CI_low, 3), CI_high = round(CI_high, 3),

`P value` = signif(p, 3)) |>

dplyr::relocate(Subgroup, level, OR, CI_low, CI_high, `P value`, `P for interaction`, Method)

}

}

dir.create("outputs/07_subgroup", recursive = TRUE, showWarnings = FALSE)

res_sbp <- purrr::map_dfr(subgroups, ~ run_one_subgroup(dat, .x, dag_covars, outcome = "pih_sbp"))

readr::write_csv(res_sbp, "outputs/07_subgroup/subgroup_primary_sbp.csv")

message("[07] Subgroup (SBP, QC) saved.")

# ============================================================

# Session info

# ============================================================

writeLines(capture.output(sessionInfo()), "outputs/session_info.txt")

message("All steps completed. See ./outputs/")

# ============================================================

# 02b) Secondary outcomes (incidence only; hospital stay = IQR)

# ============================================================

dir.create("outputs/02_secondary", recursive = TRUE, showWarnings = FALSE)

stopifnot(file.exists(path_baseline), file.exists(path_gee))

bl <- readr::read_csv(path_baseline, show_col_types = FALSE) |> janitor::clean_names()

ge <- readr::read_csv(path_gee, show_col_types = FALSE) |> janitor::clean_names()

# group standardization

std_group <- function(d){

if (!"group" %in% names(d)) stop("'group' column missing.")

if (!is.factor(d$group)) {

d <- d |>

dplyr::mutate(group = dplyr::case_when(

group %in% c("R","r","remifentanil","Remifentanil",1,"1") ~ "Remifentanil",

group %in% c("F","f","fentanyl","Fentanyl",0,"2","0") ~ "Fentanyl",

TRUE ~ as.character(group)

),

group = factor(group))

}

if (all(c("Fentanyl","Remifentanil") %in% levels(d$group)))

d$group <- factor(d$group, levels = c("Fentanyl","Remifentanil"))

d

}

bl <- std_group(bl)

ge <- std_group(ge)

# helpers

prop_ci <- function(x, n) { pt <- stats::prop.test(x, n); c(pt$conf.int[1], pt$conf.int[2]) }

qc_clip <- function(x, lo, hi){ x <- as.numeric(x); x[!is.finite(x)|x<=0] <- NA_real_; x[x<lo|x>hi] <- NA_real_; x }

find_col <- function(d, cand) { nm <- names(d); for (c in cand) if (c %in% nm) return(c); NA_character_ }

# hospital stay (median[IQR] + Wilcoxon)

cont_wilcox <- function(df, var, grp="group"){

d <- df |> dplyr::select(dplyr::all_of(c(var, grp))) |> tidyr::drop_na()

if (!is.factor(d[[grp]])) d[[grp]] <- factor(d[[grp]])

lv <- levels(d[[grp]]); x1 <- d[[var]][d[[grp]]==lv[1]]; x2 <- d[[var]][d[[grp]]==lv[2]]

fmt <- function(x){ q <- stats::quantile(x, c(.25,.5,.75), na.rm=TRUE); sprintf("%.2f [%.2f, %.2f]", q[2], q[1], q[3]) }

tibble::tibble(

Outcome = var, Test = "Wilcoxon rank-sum",

`Group 1` = fmt(x1), `Group 2` = fmt(x2), Overall = fmt(c(x1,x2)),

`P value` = sprintf("%.3f", stats::wilcox.test(x1, x2)$p.value)

)

}

# binary outcomes: n(%), p-value (Chi-square/Fisher), OR + 95% CI (Fisher)

bin_incidence <- function(df, outcome, grp="group"){

dd <- df |> dplyr::filter(!is.na(.data[[grp]])) |>

dplyr::mutate(y = as.integer(.data[[outcome]] %in% c(1,"1","yes","Yes","Y","y",TRUE)))

if (length(unique(dd[[grp]])) != 2) return(tibble::tibble())

agg <- dd |>

dplyr::group_by(.data[[grp]]) |>

dplyr::summarise(n = dplyr::n(), events = sum(y, na.rm=TRUE), prop = events/n, .groups="drop") |>

dplyr::mutate(

ci_low = mapply(function(e,n) prop_ci(e,n)[1], events, n),

ci_high = mapply(function(e,n) prop_ci(e,n)[2], events, n),

`n(%)` = sprintf("%d (%.1f%%)", events, 100*prop)

)

tab <- table(dd[[grp]], dd$y)

pval <- tryCatch({

cs <- suppressWarnings(stats::chisq.test(tab, correct=FALSE))

if (any(cs$expected < 5)) stats::fisher.test(tab)$p.value else cs$p.value

}, error = function(e) stats::fisher.test(tab)$p.value)

or_ci <- tryCatch({

ft <- stats::fisher.test(tab); c(or=unname(ft$estimate), lo=ft$conf.int[1], hi=ft$conf.int[2])

}, error = function(e) c(or=NA_real_, lo=NA_real_, hi=NA_real_))

agg |>

dplyr::mutate(Outcome = outcome) |>

dplyr::relocate(Outcome) |>

dplyr::bind_rows(

tibble::tibble(

Outcome = paste0(outcome, " (overall)"),

`n(%)` = NA_character_, !!colnames(agg)[1] := NA, n = sum(agg$n),

events = sum(agg$events), prop = NA_real_, ci_low = NA_real_, ci_high = NA_real_,

`P value` = sprintf("%.3f", pval), OR = round(or_ci["or"],3),

`95% CI (OR)` = ifelse(is.na(or_ci["lo"]), "", sprintf("%.3f-%.3f", or_ci["lo"], or_ci["hi"]))

)

)

}

# hospital stay / POC / vasopressor columns

col_hs <- find_col(bl, c("hospital_stay","length_of_stay","los"))

col_poc <- find_col(bl, c("poc","severe_cardiovascular_complication","major_cv_complication","stroke_or_mi"))

col_vaso <- find_col(bl, c("vasopressor","vasopressor_use","vasopressors","vaso_use","pressor_use"))

if (!is.na(col_hs)) bl[[col_hs]] <- suppressWarnings(as.numeric(bl[[col_hs]]))

if (!is.na(col_vaso)) bl[[col_vaso]] <- as.integer(bl[[col_vaso]] %in% c(1,"1","yes","Yes","Y","y",TRUE))

# SBP/HR extremes 0-15 min; baseline SBP

sbp_lo <- 40; sbp_hi <- 300; hr_lo <- 20; hr_hi <- 220

wide_sbp <- all(c("sbp_0","sbp_5","sbp_10","sbp_15") %in% names(ge))

wide_hr <- all(c("hr_0","hr_5","hr_10","hr_15") %in% names(ge))

if (wide_sbp) {

ge$sbp_0 <- qc_clip(ge$sbp_0, sbp_lo, sbp_hi)

ge$sbp_5 <- qc_clip(ge$sbp_5, sbp_lo, sbp_hi)

ge$sbp_10 <- qc_clip(ge$sbp_10, sbp_lo, sbp_hi)

ge$sbp_15 <- qc_clip(ge$sbp_15, sbp_lo, sbp_hi)

ge <- ge |>

dplyr::rowwise() |>

dplyr::mutate(sbp_max_0_15 = {v <- c_across(c(sbp_0,sbp_5,sbp_10,sbp_15)); if (all(is.na(v))) NA_real_ else max(v, na.rm=TRUE)}) |>

dplyr::ungroup()

}

if (wide_hr) {

ge$hr_0 <- qc_clip(ge$hr_0, hr_lo, hr_hi)

ge$hr_5 <- qc_clip(ge$hr_5, hr_lo, hr_hi)

ge$hr_10 <- qc_clip(ge$hr_10, hr_lo, hr_hi)

ge$hr_15 <- qc_clip(ge$hr_15, hr_lo, hr_hi)

ge <- ge |>

dplyr::rowwise() |>

dplyr::mutate(hr_min_0_15 = {v <- c_across(c(hr_0,hr_5,hr_10,hr_15)); if (all(is.na(v))) NA_real_ else min(v, na.rm=TRUE)}) |>

dplyr::ungroup()

}

if (!wide_sbp || !wide_hr) {

need <- c("patient_id","time","sbp","hr")

if (!all(need %in% names(ge))) stop("Require patient_id/time/sbp/hr for long format.")

ge_long <- ge |> dplyr::select(patient_id, time, sbp, hr) |> dplyr::filter(time %in% c(0,5,10,15))

ge_long$sbp <- qc_clip(ge_long$sbp, sbp_lo, sbp_hi)

ge_long$hr <- qc_clip(ge_long$hr, hr_lo, hr_hi)

sbp_max <- ge_long |> dplyr::group_by(patient_id) |> dplyr::summarise(sbp_max_0_15 = if (all(is.na(sbp))) NA_real_ else max(sbp, na.rm=TRUE), .groups="drop")

hr_min <- ge_long |> dplyr::group_by(patient_id) |> dplyr::summarise(hr_min_0_15 = if (all(is.na(hr))) NA_real_ else min(hr, na.rm=TRUE), .groups="drop")

ge <- ge |> dplyr::left_join(sbp_max, by="patient_id") |> dplyr::left_join(hr_min, by="patient_id")

}

if (!"sbp_base" %in% names(ge)) {

if ("sbp_0" %in% names(ge)) ge$sbp_base <- ge$sbp_0 else {

sbp0 <- if (exists("ge_long")) ge_long |> dplyr::filter(time==0) |> dplyr::select(patient_id, sbp0=sbp) else NULL

if (!is.null(sbp0)) ge <- ge |> dplyr::left_join(sbp0, by="patient_id") |> dplyr::mutate(sbp_base = sbp0)

}

}

if ("sbp_base" %in% names(ge)) ge$sbp_base <- qc_clip(ge$sbp_base, sbp_lo, sbp_hi)

ge <- ge |>

dplyr::mutate(

hyper_sbp = dplyr::case_when(

is.finite(sbp_max_0_15) & is.finite(sbp_base) ~ as.integer(sbp_max_0_15 > 180 | (sbp_max_0_15 - sbp_base)/sbp_base >= 0.20),

is.finite(sbp_max_0_15) & !is.finite(sbp_base) ~ as.integer(sbp_max_0_15 > 180),

TRUE ~ NA_integer_

),

brady_hr = dplyr::if_else(is.finite(hr_min_0_15), as.integer(hr_min_0_15 < 50), NA_integer_)

)

# compute tables

sec_dir <- "outputs/02_secondary"

hs_tbl <- if (!is.na(col_hs)) cont_wilcox(bl, col_hs, "group") else tibble::tibble()

poc_tbl <- if (!is.na(col_poc)) bin_incidence(bl, col_poc, "group") else tibble::tibble()

hyper_tbl<- if ("hyper_sbp" %in% names(ge)) bin_incidence(ge, "hyper_sbp", "group") else tibble::tibble()

brady_tbl<- if ("brady_hr" %in% names(ge)) bin_incidence(ge, "brady_hr", "group") else tibble::tibble()

if (is.na(col_vaso)) {

col_vaso_ge <- find_col(ge, c("vasopressor","vasopressor_use","vasopressors","vaso_use","pressor_use"))

if (!is.na(col_vaso_ge)) ge[[col_vaso_ge]] <- as.integer(ge[[col_vaso_ge]] %in% c(1,"1","yes","Yes","Y","y",TRUE))

vaso_tbl <- if (!is.na(col_vaso_ge)) bin_incidence(ge, col_vaso_ge, "group") else tibble::tibble()

} else {

vaso_tbl <- bin_incidence(bl, col_vaso, "group")

}

if (nrow(hs_tbl)) readr::write_csv(hs_tbl, file.path(sec_dir, "hospital_stay_by_group.csv"))

if (nrow(poc_tbl)) readr::write_csv(poc_tbl, file.path(sec_dir, "poc_by_group.csv"))

if (nrow(hyper_tbl)) readr::write_csv(hyper_tbl, file.path(sec_dir, "hypertension_by_group.csv"))

if (nrow(brady_tbl)) readr::write_csv(brady_tbl, file.path(sec_dir, "bradycardia_by_group.csv"))

if (exists("vaso_tbl") && nrow(vaso_tbl)) readr::write_csv(vaso_tbl, file.path(sec_dir, "vasopressor_use_by_group.csv"))

sheets <- list()

if (nrow(hs_tbl)) sheets[["Hospital_stay"]] <- hs_tbl

if (nrow(poc_tbl)) sheets[["POC"]] <- poc_tbl

if (nrow(hyper_tbl)) sheets[["Hypertension_SBP"]] <- hyper_tbl

if (nrow(brady_tbl)) sheets[["Bradycardia_HR"]] <- brady_tbl

if (exists("vaso_tbl") && nrow(vaso_tbl)) sheets[["Vasopressor_use"]] <- vaso_tbl

if (length(sheets)) writexl::write_xlsx(sheets, path = file.path(sec_dir, "secondary_outcomes.xlsx"))
